# Memory, innovation and vertical learning

**DOI:** 10.1371/journal.pcbi.1013785

**Published:** 2025-12-05

**Authors:** Madeleine Ammar, Laurel Fogarty, Anne Kandler

**Affiliations:** Theory in Cultural Evolution Lab, Department of Human Behavior, Ecology, and Culture, Max Planck Institute for Evolutionary Anthropology, Leipzig, Germany; Dartmouth College Department of Psychological and Brain Sciences, UNITED STATES OF AMERICA

## Abstract

One of the most controversial and actively debated questions about both human and non-human animal culture concerns its relationship with adaptation – under what circumstances might we expect culture, and the ability to learn socially from others, to be beneficial, and favored by natural selection? Existing theory posits that the benefit of social learning depends on the rate at which the environment changes, and recent work has shown that this relationship is mediated by how much information an individual can retain over time - by memory. Based on extensive ethnographic research, vertical learning—social learning from parent to offspring—appears to be an extremely salient and important type of social learning. Here we develop large-scale agent-based simulation models to investigate the evolutionary relationship between vertical social learning in particular and the memory and retention of cultural information.

We show that the benefit of vertical learning depends on how quickly those information is forgotten and on the exact way in which individuals innovate. This work points to the importance of a complex interplay between the size of cultural repertoires, the benefits of cultural preservation, changing selective environments, and the mechanism of innovation - none of which can be fully understood in isolation.

## Author summary

One of the most debated questions about both human and animal culture is when it is beneficial and supported by evolution? Existing theory posits that this depends on the speed of environmental change, and new research shows that memory, or how much information someone can hold, plays a big role, too. Ethnographic studies indicate that learning from parents (known as vertical learning) is a particularly important form of cultural learning. In this study, we use large-scale simulations to explore the interplay between vertical learning and memory in the retention of cultural knowledge over time. Our findings show that the advantage of vertical learning depends on how quickly individuals forget information and how they come up with new ideas through innovation. This highlights the complex interaction between the environment, the amount of cultural knowledge both held by the individual and transmitted across generations, and the process of innovation.

## Introduction

Social learning, defined as ‘learning that is influenced by observation of, or interaction with [...] a conspecific or its products’ [1], is widespread in the animal kingdom [2] and a seemingly extraordinary capacity for complex forms of social learning [3] is often credited for human demographic success. The evolution of social learning and cultural transmission, therefore, has been a target of research for decades (e.g., [2,4–12]). Much of this work has focused on understanding the spread of behaviours, which are passed to naïve individuals either innately (or genetically) or socially, or which are newly innovated.

The transmission of behaviours directly from parents to offspring, a type of social learning known as vertical transmission [6,11,13] or vertical learning [14], appears to be particularly important in the context of human learning and human cultural evolution [16]. Hewlett & Cavalli-Sforza [16] investigated the source of a large number of technologies and behaviours in the Aka, a foraging population living in the Central African Republic, and concluded that over 80% of the cultural traits or behaviours possessed by individuals in these populations were transmitted vertically, or in other words, learned from biological parents. A similar study of the sources of beliefs and habits of Stanford undergraduates came to similar conclusions [17], although such estimates are the subject of some controversy (see e.g., [14,15]).

The circumstances under which vertical learning is adaptive are, nevertheless, likely to be important to our understanding of the evolution of human social learning in particular. Theoretical models have shown that in an unchanging constant environment, vertical learning increased the speed at which beneficial phenotypes could fix in a population compared to spread via oblique social learning, i.e. the transmission of behaviour from an older (unrelated) individual to a younger individual [18]. When the environment instead changed rapidly, they showed that vertical learning was disfavoured compared to oblique social learning. Similar results were obtained by McElreath & Strimling [14] who suggested that environmental stability was among the important forces driving the evolution of vertical learning.

These results echo the findings from work that contrasts the adaptive value of innate transmission of behaviour, social learning, and innovation. This work showed that innate transmission was favoured by natural selection when the rate of environmental change was very slow, social learning was favoured at intermediate rates of environmental change, and that high rates of innovation were favoured when the environment changed rapidly [6].

One simplifying assumption common in work on this topic is that an individual can engage in vertical learning, oblique social learning, or innovation in order to adapt to their environments, only once in their lifetimes. Further, many such models assume that individuals remember just one behaviour at a time. Here, the adoption of a new behaviour implies forgetting the one used before. Ethnographic evidence, however, suggests that enculturation, or culture learning, in many human populations is more complex. For example, Aunger [15] studied several populations of horticulturalists and foragers in the Central African Republic and suggested that their learning was phased with a strong emphasis on vertical learning early in life, and a strong emphasis on oblique social learning later in life. Similarly, Hewlett et al. [19] showed that individuals amassed cultural information in a way that was phased across life stages in Congo Basin hunter gatherers, and that the timing of the phases of different types of cultural learning showed some cross-cultural variation.

In a recent study [20] we considered a situation where an individual first obtains a repertoire of cultural variants from their parent, and then has the capacity to add to, or subtract from, this cultural repertoire throughout its lifetime. In particular, we focused on the evolution of social learning in temporally changing, recurring environments by allowing for the interplay between social learning, in form of payoff-biased social learning, and innovation, alongside a capacity for ‘memory’, i.e., the storage, retrieval, and forgetting of information. Individuals acquire cultural variants by innovation or social learning, maintain a bounded set in their repertoires, and sample and express traits from that repertoire. Memory decouples the immediate value of a cultural variant from its “long-term” value. That buffering favours social learning in broad range of environmental conditions: higher propensities for social learning can produce faster population-level adaptation since variants adapted to alternative environmental states can be preserved without affecting individual fitness and be expressed once states recur. At the same time, the strength of forgetting evolves to prevent the unbounded accumulation of especially low-benefit variants, producing repertoires whose sizes and compositions are tuned to the environmental stability experienced by the population. Thus, the composition of the cultural repertoires, i.e. the number of cultural variants and their adaptation values, is an outcome of the evolutionary dynamics.

Here, we build on this model to investigate the role that vertical learning plays in adaptation to changing environments, allowing individuals to engage in vertical learning, oblique or horizontal social learning and innovation throughout their lifetimes. This flexible model allows for the accumulation of cultural information from different sources, provides a ‘role for individuals in their own education’, as suggested by [15], and brings a focus on processes involved in remembering and forgetting cultural information.

The precise mechanism of innovation might also affect the evolution of social and vertical learning. Models exploring the evolution of social learning have formulated innovation in a number of ways. For example, it is often assumed that individuals can interact with the environment and, through ‘trial-and-error’ learning, immediately discover a behaviour that matches the environment well. Often, although such individuals will be well adapted to the environment, they will also incur a cost for trial-and-error learning (e.g., [10,21]). Such costs reflect the dangers of such experimentation or, alternatively, the time involved. The implicit assumption that good information is readily available through innovation means that, in very changeable environments, relying on innovation rather than social learning or even vertical learning is reasonable. However, if good information is, instead, very difficult to find and innovation is relatively ineffective, reliance on innovation would only be reasonable across a narrow range of environmental stability parameters. Here, we investigate the interaction between innovation effectiveness and the evolution of social and, in particular, vertical learning.

In agreement with previous work, our model shows that vertical learning is advantageous when the environment is relatively stable and disadvantageous when the environment changes extremely rapidly. However, our model also suggests that vertical learning is *most* advantageous where environmental change happens at intermediate rates. The relative benefits of vertical learning at intermediate rates of environmental change depend on the process of innovation and how cultural variants are maintained. Where innovation is easy, any information that is lost can be replaced when needed. In this case, vertical learning, which can maintain this information, is useful but not essential. However, when innovation is ineffective and replacing lost information takes many timesteps, vertical learning provides a larger advantage.

## 1 Methods

The model used in the following is built on [20] with the main difference that (i) vertical learning can be present or absent (see Sect 1.4) and (ii) different models of innovating new cultural variants have been implemented (see Sect 1.2.2). For convenience of the reader we redescribe the modelling details in this section.

### 1.1 Simulation framework

We consider an age-structured population of *N* individuals that experience a temporally varying environment, *E*(*t*). The environment fluctuates between two distinct states E(t)∈{1,2} with probability $p_{\text{env}}$ at every time step *t* implying that the average waiting time between two environmental changes is given by $T_{\text{change}}=1/p_{\text{env}}$. Individuals are characterised by the cultural variants they have adopted and their genetically transmitted propensity to engage in social learning, ξ, (see Sect 1.2.2) and their probability to forget cultural information, φ, (see Sect 1.3).

A cultural variant is characterized by its adaptation value, *a*(*k*), representing the level of benefit the variant conveys to its adopter in environmental state *k*. Adaptation values can range between 0 and 1 (with 1 denoting the maximum and 0 the minimum). We assume that a variant can only be adapted to a single environmental state, i.e. if a variant possesses an adaptation value greater than 0 in one environment it possesses an adaptation value of 0 in the other. These two states may correspond to coarse categories like “hot” and “cold” or “wet” and “dry”. Any environmental change will thus have an effect on the benefit of a variant’s expression depending on its adaptation value in the current state. This dynamic will ultimately influence the evolution of the rates of learning and forgetting.

Individuals learn about the level of benefit a cultural variant conveys in environmental state *k* through social learning or innovation (see Sect 1.2.2). As we assume that individuals can accurately infer in which environmental state they currently exist, this will generate a precise mapping between environmental state and level of benefit. Importantly, individuals can maintain knowledge about a number of cultural variants. The adaptation value of every socially learnt or innovated cultural variant of individual *j* is stored in its cultural repertoires, *M^j^*, at least temporally. The number of variants in the cultural repertoire, *m^j^*, can reach 500 but it can never fall below 1. This way, an individual, *j*, must always know about at least one variant and its repertoire, *M^j^*, can never be empty.

### 1.2 Expression dynamic

Each time step, all individuals must express a variant whose adaptation value determines their individual fitness levels. For this purpose, individuals make an informed decision based on their cultural repertoire. First, they evaluate their cultural repertoire and choose a variant from it. Second, depending on the adaptation value of this chosen variant they either express it or, if the chosen variant is deemed not beneficial enough, engage in social learning or innovation. The decision to learn or innovate is controlled by the genetically transmitted propensity for social learning, ξ. The learnt or innovated variant is then expressed, regardless of the adaptation value and, if not present already, added to the cultural repertoire. In the following we provide a detailed description of these decisions.

#### 1.2.1 Choice of cultural variant from individual repertoire.

An individual, *j*, chooses variant *i* from its repertoire, *M^j^*, with a probability proportional to the variant’s adaptation value, i.e. the conveyed benefit, in the current environment, *a*_*i*_(*k*), relative to the rest of the repertoire

$$p_{i}^{j}(k)=\frac{a_{i}(k)}{\sum_{s=1}^{m^{j}}a_{s}(k)}.$$ (1)

The rationale behind decision rule (1) stems from the tendency of human decision-making to be error-prone, which means that individuals might not consistently select the option with the highest adaptation value from the available choices for various reasons. Additionally, we investigate the impact of an alternative decision rule frequently examined in optimal decision-making contexts, specifically the softmax rule ([22], p. 5). The results of this exploration are detailed in S4 Sect of the supplementary material.

Based on the adaptation value of the chosen variant *i*, individual *j* decides whether to express variant *i* or not: with probability *a*_*i*_(*k*) individual *j* expresses *i*, and with probability $1\;-\;a_{i}(k)$ *j* decides to engage in social learning or innovation. Put differently, a high adaptation value of the chosen variant will prevent learning or innovation in this time step.

#### 1.2.2 Learning.

If individual *j* has decided to not express the variant chosen from its repertoire it engages in social learning with probability $\xi_{j}$ and in innovation with probability $1-\xi_{j}$.

- **Social learning:** An individual *j* observes the levels of benefit gained by all individuals during the last time step and chooses to copy a variant proportional to its observed benefit. In other words, it engages in payoff-biased social learning (e.g., [2]). Then individual *j* expresses this learned variant, *x*, receives the benefit *a*_*x*_(*k*), and adds *x* to its repertoire *M^j^* (if it is not already contained in it).
- **Innovation:** In general, innovation events introduce new variants into the system. Here we focus on the impact of three different innovation processes that differ in the probability with which highly adaptive variants can be introduced. Two further innovation processes where we alter the assumption about the benefit provided in the alternative environment, are described in the supplementary material (see S5 Sect), which are referenced where informative.
  – Type I innovations: The adaptation value of the new variant is uniformly chosen from the interval [0,1].
  – Type II innovations: With a probability of 0.95 the adaptation value of the new variant is uniformly chosen from the interval [0,0.8] and with probability 0.05 from the interval [0,1]. This implies that innovations of highly adaptive cultural variants are rare and the vast majority of the innovations provide a low/medium benefit.
  – Type III innovations: With a probability of 0.95 the adaptation value of the new variant is close to zero, i.e. the innovation effectively failed, and with probability 0.05 it is chosen from the interval [0,1]. As for type II innovation, introducing highly adaptive cultural variants are rare but now the vast majority of innovations provide no benefit.
  Individual *j* expresses the innovated variant, *x*, receives the benefit *a*_*x*_(*k*), and adds *x* to its repertoire *M^j^*.

Last, we track how often a variant *i* has been expressed by individual *j* and denote this number of expressions by $n_{i}^{j}$.

### 1.3 Forgetting

In each time step, individuals can lose knowledge. $\varphi_{j}$ defines the probability that individual *j* removes one cultural variant from its repertoire *M^j^* with the restriction that the variant expressed in the current time step cannot be forgotten, i.e. the minimum size of the repertoire is 1. Rarely expressed variants are more likely to be forgotten compared to frequently used variants. More precisely, if individual *j* “decides” to forget, it “chooses” variant *z* to be removed from its repertoire with probability

$$f_{z}^{j}=\frac{A_{j}-n_{z}^{j}}{\sum_{\begin{array}{c} s=1\\ s\neq i \end{array}}^{m^{j}}(A_{j}-n_{s}^{j})},$$ (2)

where $A_{j}=\sum_{\begin{array}{c} s=1\\ s\neq i \end{array}}^{m^{j}}n_{s}^{j}$ and *i* denotes the variant expressed in this time step that can never be forgotten. We note that Eq (2) is not the only plausible choice for a forgetting rule (see [20], for a discussion of alternative choices).

### 1.4 Birth-death process and vertical learning

In each time step, one individual is chosen for reproduction and one for death. The reproducing individual is chosen proportionally to the benefit generated in this time step, i.e. individual *j* is chosen with probability

$$v_{j}(t)=\frac{a_{j}(t)}{\sum_{s=1}^{N}a_{s}(t)}.$$ (3)

The process of death is age-dependent, i.e. the probability that an individual dies is proportional to its relative age such that older individuals are more likely to die than younger individuals. This way, we ensure that individuals do not become unreasonably old to isolate the effects of vertical learning from the preservation of cultural variants through the long life spans of some individuals. In the supplementary material, we demonstrate that the age distribution of populations experiencing age-dependent death is narrower than for a conventional Moran model with random death (see S1 Sect). If individual *j* is chosen for reproduction, it passes its ξ- and φ-value to its offspring. This happens faithfully with a probability $1-\mu_{\xi }$ and $1-\mu_{\varphi }$, respectively. Put differently, with probability $\mu_{\xi }$, the offspring will possess


$$\xi_{j}+\varepsilon \;\text{with}\;\varepsilon ∼𝒩(0,\sigma_{\xi }^{2}).$$

and with probability $\mu_{\varphi }$


$$\phi_{j}+\varepsilon \;\text{with}\;\varepsilon ∼𝒩(0,\sigma_{\varphi }^{2}).$$

Note that the normally distributed errors are truncated to avoid values larger than 1 or smaller than 0. To investigate the importance of vertical learning, in particular for adaptation, we consider two scenarios. In the first scenario, we do not allow for vertical learning, i.e. offspring are born without knowledge about any cultural variant and individuals have to acquire their first variants through social learning or innovation. In the second scenario, we assume that offspring inherit the entire cultural repertoire of their parent but it does not inherit information about the parent’s experiences of the variants in the repertoire—the $n_{i}^{j}$-values describing the number of times variant *i* was expressed by parent *j*. Thus, at first, all vertically learned variants are equally susceptible to forgetting as their numbers of expressions are set to 1. Contrasting these extreme scenarios has the advantage of revealing the strongest effects of vertical learning. Our definition of vertical learning is in line with others (e.g., [6,13,23]), with repertoires consisting of more than a single variant due to the capacity for memory.

### 1.5 Simulation set-up

We consider a population of *N* = 200 individuals. Each simulation consists of a burn-in phase of 50 000 time steps and is followed by another 50 000 time steps, thus the total simulation time equals 500 generations. Initially, both the propensity for social learning, ξ, and the probability to forget, φ, are set to 0.2 for all individuals. Fixed parameters include $\mu_{\xi }=0.05$, $\sigma_{\xi }^{2}=0.1$, $\mu_{\varphi }=0.05$, $\sigma_{\varphi }^{2}=0.1$. For any given set of parameters we ran 3000 independent simulations.

## 2 Results

By definition, the presence or absence of vertical learning determines the way in which naive individuals acquire their first cultural variants. In populations that do not engage in vertical learning, naive individuals obtain their first cultural variant either through social learning or innovation. If the environment remains unchanged, the adaptation value of this first variant determines the likelihood of further learning or innovation events. For example, a high adaptation value implies a low likelihood of future learning or innovation events and the opposite is true for a low adaptation value (see Sect 1.2.2).

Without vertical learning, the first environmental change an individual experiences will always result in a learning or innovation event as no cultural variant adapted to the changed environmental conditions can be contained in its repertoire. For all subsequent environmental changes an individual can either rely on its repertoire or engage in learning or innovation. Thus, the quality of the available social information and the nature of the innovation process determine, in part, the size of the individual repertoires. As we show below, the absence of vertical learning leads to relatively low average repertoire sizes. In contrast, vertical learning results, on average, in large individual repertoires when the environment is unstable and small repertoires when it is stable [20].

Particularly, in unstable environments when individuals are likely to experience multiple environmental changes in their lifetimes, the low average repertoire sizes are in stark contrast to those expected in populations that do engage in vertical learning. Such populations have been shown to produce relatively large and highly structured repertoires [20]. By “structure” we mean the shape of the distribution of adaptation values within individual repertoires. Highly structured repertoires contain a large proportion of highly adaptive variants, i.e., the distribution of adaptation values is strongly skewed toward 1. Such structures arise partly from vertical learning of whole repertoires combined with low forgetting rates, which buffer against the stochastic loss of highly adaptive variants in either environmental state and thereby facilitate adaptation from standing, unexpressed variation. Additionally, the specifics of the way individuals choose a variant from their repertoires influence the development of the structure of the individual repertoires. For example, rule (1) imposes pressure on the repertoires to become structured because only such structures yield the choice of a highly adaptive variant on average. Alternative decision rules that are less sensitive to repertoire structure could alter the evolutionary dynamics by reducing the dependence on highly structured repertoires (see S4 Sect).

In more stable environments, however, populations with and without vertical social learning evolve higher rates of forgetting (see Figs A, B, and C in S3), and as a result are characterized by similar average individual repertoire sizes. Nevertheless, we observe differences in the composition of these repertoires. Without vertical learning, cultural variants adapted to the alternative environment are lost at the very latest after the death of the last individual that experienced this alternative environment. The intergenerational transmission facilitated by vertical learning means these variants can be found in the repertoires of individuals that have not experienced the alternative environment, effectively prolonging the lifetime of information about the alternative environment.

**Fig 1:**
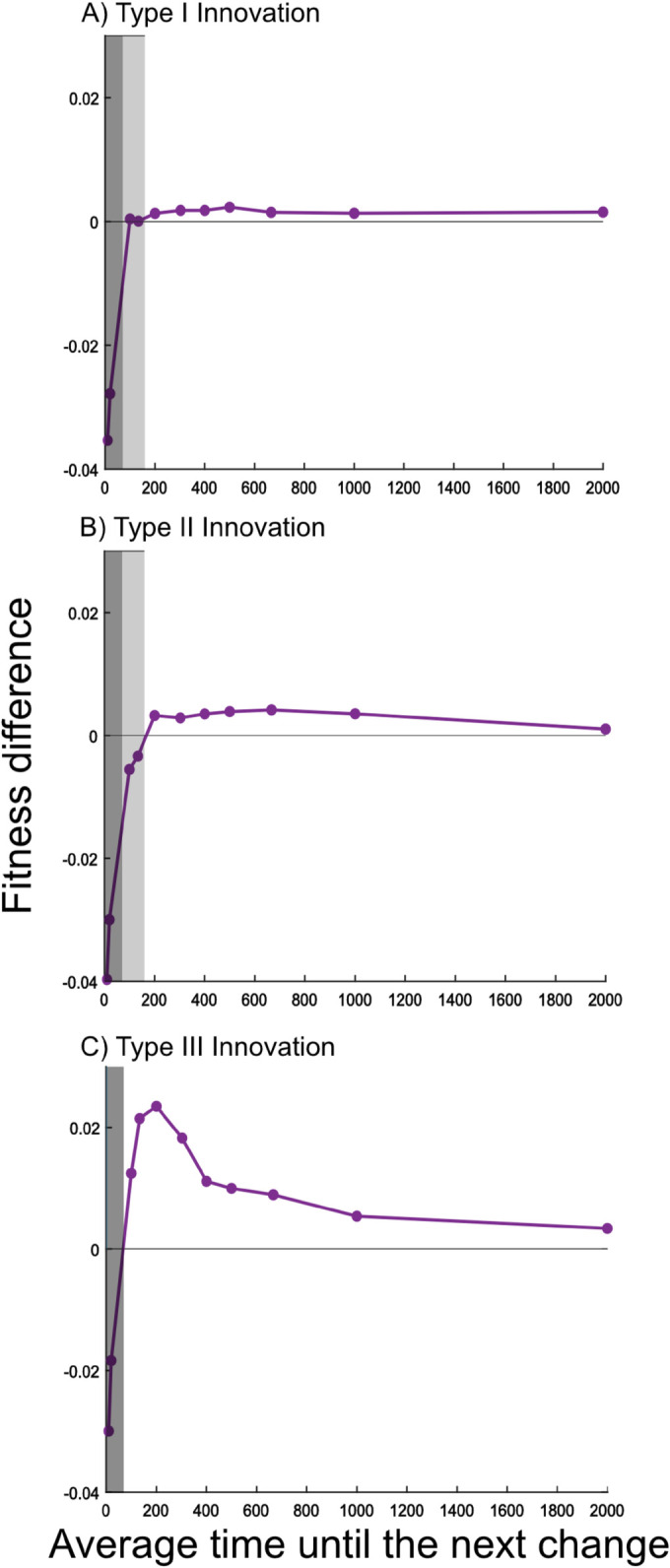
Advantage of vertical learning. Relationship between environmental variability and average fitness difference between populations with and without vertical learning. Values are calculated as the average expression value of variants by all individuals in the last generation (i.e. 200 time steps) of a simulation. Fitness values of populations without vertical learning are subtracted from populations exhibiting vertical learning. Population averages are further averaged over all 3000 simulations. Dark grey boxes represent areas, where vertical learning is disadvantageous, white boxes represent areas with a benefit of vertical learning, light grey boxes, where present, indicate intermediate dynamics. The three panels represent the three innovation regimes.

In summary, besides influencing the way in which naive individuals acquire their first cultural variant, the presence or absence of vertical learning affects (i) the size and structure of the individual repertoires, and (ii) the persistence of information about the alternative environment differently in different environmental scenarios. These differences dictate whether vertical learning is advantageous for the population. To quantify the advantage or disadvantage of vertical learning we show in Fig 1 the difference in the average fitness level of populations engaging and not engaging in vertical learning for all innovation types. Negative values indicate that vertical learning leads, on average, to a lower fitness level. The reverse is true for positive values.

**Fig 2:**
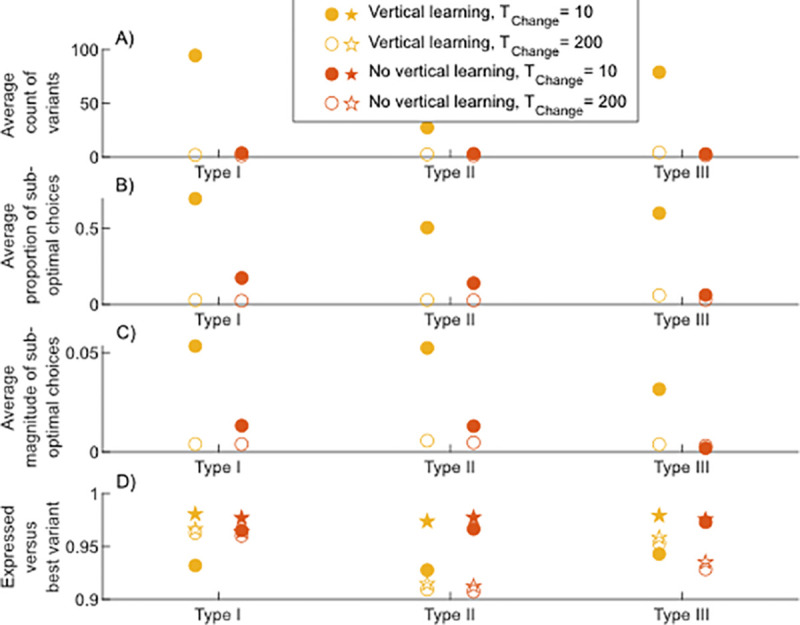
Repertoire size and features of sub-optimal variant choice. Results are shown for different $T_{\text{change}}$ values (*x*-axis) and three innovation regimes. Filled dots correspond to $T_{\text{change}}$ = 10, open dots correspond to $T_{\text{change}}$ = 200. A) Average count of variants: Values are calculated as the average variant count per individual in the last time step of a simulation. B) Average proportion of individual’s sub-optimal variant: Values are calculated as the age-normalized proportion of sub-optimal variant choices of individuals living in the last generation of a simulation. C) Average magnitude of sub-optimal choice: Values are calculated as the age-normalized average distance between individual’s sub-optimal variant choice and the best variant in their repertoire during the last generation of a simulation. D) Average adaptive value of expressed variants (dot) versus the best variant (star) in an individual’s repertoire: Values are calculated for the last time step of a simulation.

In general, the absence of vertical learning proves advantageous in unstable environments because the smaller cultural repertoires contain cultural variants well-adapted to both environmental states which allows for consistently better variant choices (see Fig 2). At the same time information about the alternative environmental state is kept in the population with certainty.

In more stable environments the presence of vertical learning proves advantageous. In this case, the average size of the individual repertoires is similar to the case of no vertical learning but information about the alternative environment persists for longer in the population facilitating adaptation through standing, unexpressed variation (see Fig 4). Intuitively, the probability of preserving information about the alternative environment until the next environmental change decreases with increasing environmental stability, even when forgetting is low.

In very stable environments vertical learning provides a small advantage that is not ascribed to the preservation of information. During the long periods of stasis, parents transmit their highly adaptive cultural variants and thus, their offspring do not engage in other forms of learning to acquire their initial cultural variants avoiding the introduction of low-benefit variants through, for example, innovation.

To aid interpretation of the results we define two parameter ranges (see dark grey and white areas in Fig 1) that indicate whether the differences between the cultural dynamic in populations with and without vertical learning are primarily caused by (i) or (ii) defined above.

However, we note that the distinction between the two ranges is not clear-cut (see e.g., light grey areas in Fig 1). There exist some intermediate rates of environmental change where the dynamics underlying adaptation balance, and both factors—the size of the repertoire and the persistence of information—play an important role. In the following sections we detail how a balance between dynamics dominated by (i) or (ii) affects the role of vertical learning in the adaptation process when innovation operates in different ways.

**Fig 3:**
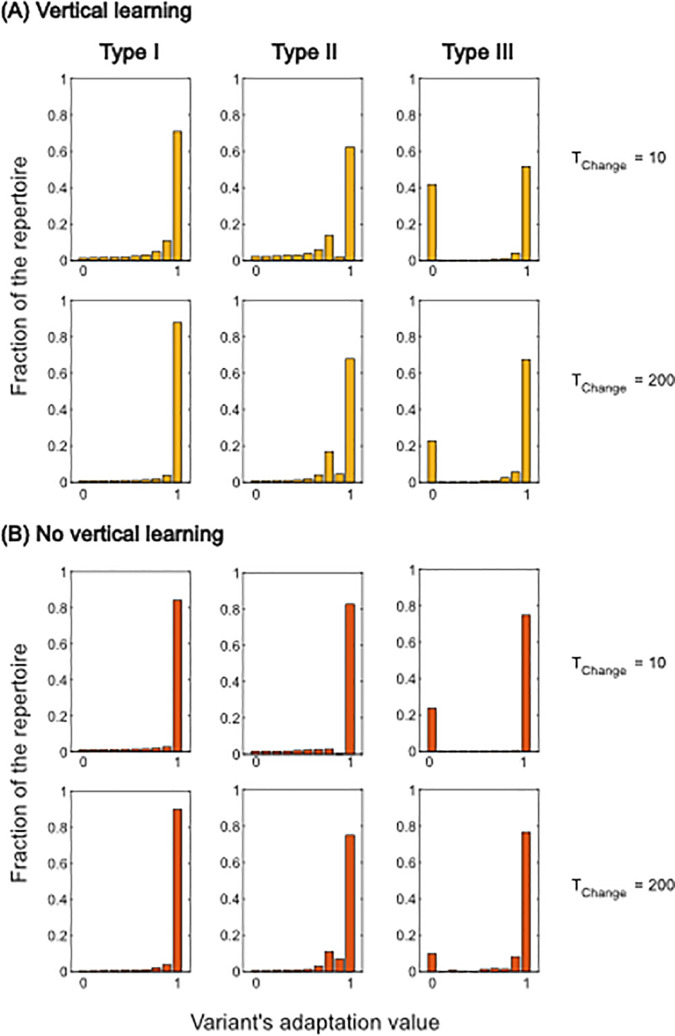
Cultural repertoires. Repertoire composition at different $T_{\text{change}}$ values ($T_{\text{change}}$ = 10 in 1^*st*^ and 3^*rd*^ row; $T_{\text{change}}$ = 200 in 2*^nd^* and 4^*th*^ row) with vertical learning (top panels, in yellow) and no vertical learning (lower panels, in red). Each bar shows the fraction of cultural variants that fall within a given adaptation interval, ranging from 0 (lowest adaptation value, left) to 1 (highest adaptation value, right). Fractions refer to variants adapted to the current environment. Fractions are calculated for single repertoires and further averaged over all simulations. The three columns represent the three innovation regimes.

We quantify the size and structure of the individual repertoires by recording the average number of variants adapted to each environmental state along with the number of sub-optimal variants that individuals choose to express from their repertoires, i.e. the fraction of times individuals do not choose the variant with the highest adaptation value available to them from their repertoire, the magnitude of sub-optimal choices, i.e. the average distance of a sub-optimal variant choice from the best variant in an individual’s repertoire (see Fig 2B and 2C), and the average adaptive values of variants that are actually expressed by individuals alongside their best variants (Fig 2D). Additionally, we illustrate the overall structure of the individual repertoires by plotting the distributions of the adaptation values of all cultural variants contained in the population (see Fig 3 and for more details A, B, and C in S2).

**Fig 4:**
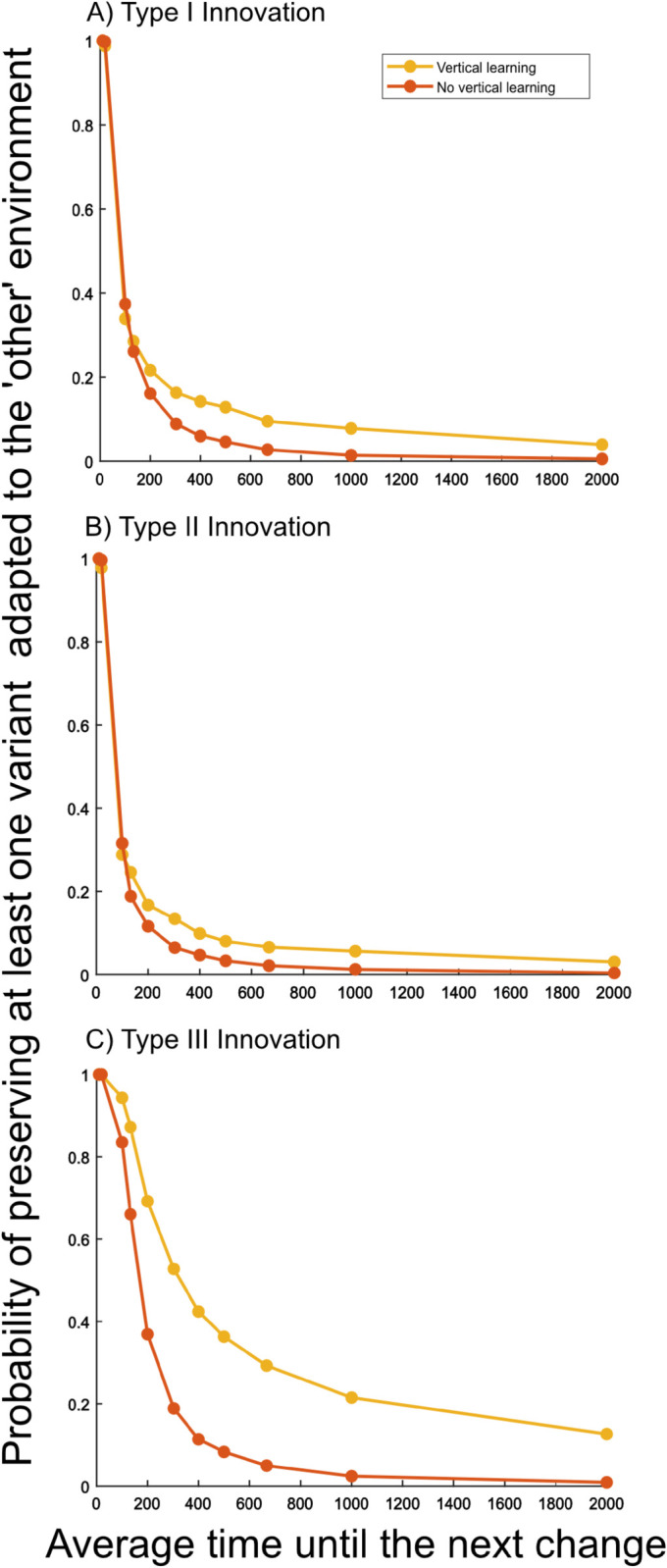
Preservation of unexpressed variants. Relationship between environmental variability and the probability of preserving at least one variant adapted to the ‘alternative’ environment until the next change occurs. Simulations are run until the burn-in phase and stopped once the first change occurs (before learning takes place). For each simulation it is recorded whether the population consists of at least one individual that knows about a variant adapted to the ‘alternative’ environment. Values are averaged over all 3000 simulations. Yellow lines correspond to simulations with vertical learning, red lines correspond to simulations without vertical learning. The three panels represent the three innovation regimes.

To quantify the persistence of information about the alternative environment, and so the potential of adaptation through unexpressed standing variation, we calculate the probability that at least one individual in the population possesses a variant adapted to the alternative environment at the point of an environmental change (see Fig 4).

### 2.1 Type I innovations

#### 2.1.1 Repertoire size and structure.

When the rate of environmental change is high, i.e. $T_{\text{change}}<100$, or put differently, when individuals are likely to experience multiple environmental changes in their lifetime, vertical learning is, on average, disadvantageous (see Fig 1A, dark grey area).

In this range, populations with and without vertical learning have the same potential for adaptation through unexpressed standing variation—Fig 4A shows that in both scenarios there is typically at least one individual in the population that has a cultural variant adapted to the alternative environmental state. Together with high, and similar, propensities for social learning (see Fig A in S3), this implies that adaptation to changed environmental conditions proceeds mainly through the expression of highly adaptive variants chosen from individual repertoires and their subsequent spread through the process of social learning.

However, Fig 2 shows that populations with vertical learning more often choose to express variants with an adaptation value lower than the maximum in their repertoires compared to populations with no vertical learning (see Fig 2D). In other words, individuals are more likely to choose a variant from their repertoire that is not their best (see Fig 2B) and the deviation from the best variant is more severe (see Fig 2C). This is caused by their relatively large individual repertoires which contain more variants with low adaptation value compared to the case of no vertical learning (see Figs 3A and A in S2; for an explicit demonstration of the relationship between increasing repertoire size, changing distributions of variants and variant choice, see Fig E in S2). This results in lower average fitness levels for populations with vertical learning compared to populations without in variable environments (see Fig A in S3). We note that the effect of the repertoire size and structure on fitness, depends on the exact formulation of the variant choice rule. When individuals employ a less error-prone rule, sub-optimal choices, although equally frequent, are less severe (compare Figs D in S2 and C in S4) (for more information see supplementary material S4 Sect). This reduces the disadvantage of vertical learning in variable environments (see Fig A in S4).

#### 2.1.2 Standing variation.

When the rate of environmental change is low, i.e. $T_{\text{change}}\geq 200$, vertical learning has a small but consistently positive effect on adaptation (see white region in Fig 1A). Figs 2A and 3 show that, in this parameter range, the individual repertoires for populations with and without vertical learning are, on average, small and highly structured, owing to high rates of forgetting (see Fig A in S3). This results in a reduction in the average frequency and magnitude of sub-optimal choices from these repertoires for both vertical and non-vertical learners (see Fig 2B and Fig 2C). This is also seen in Fig 2D where the stars showing the best available variants overlap with the dots showing the expressed variants.

More important here is the transgenerational transmission of information by vertical learning. This preserves cultural variation, which remains unexpressed but present in repertoires for a longer period of time compared to situations with no vertical learning. In general, when an environmental change occurs, the presence of variants adapted to the alternative environment in the cultural repertoire of at least one individual, circumvents the need to wait for innovation to introduce a new adaptive variant—they allow for adaptation from standing variation. Fig 4A shows the probability that at least one individual carries a cultural variant that is adapted to the alternative environment in their repertoire. This probability is higher in populations with vertical learning, compared to populations without. As a consequence, populations with vertical learning have slightly higher average fitness levels for these relatively stable environmental conditions. The small magnitude of these differences shown in Fig 1A is rooted in the interaction between the innovation process and the evolution of forgetting rates.

For $T_{\text{change}}=100,133$, populations with and without vertical learning lead to very similar average fitness levels (see light grey region in Fig 1A). Here, we see that both, the frequency of sub-optimal choices of expressed variants and the probability that at least one individual has information adapted to the alternative environment at the point of an environmental change is similar for populations of vertical learners compared to those of non-vertical learners (see Figs D in S2 and 4A). Both forces—one leading to an advantage for populations with vertical learning and one leading to an advantage for populations without vertical learning—are balanced.

Under a less error-prone variant choice rule the advantage of vertical learning in relatively stable environments is further increased (see Fig A in S4). This is due to the relaxed pressure on forgetting to evolve to high rates rates (compare Figs A in S3 and D in S4) which maintains variants adapted to the alternative environment longer in the individual repertoires (see Fig E in S4).

### 2.2 Type II innovations

To explore how the results described in the previous sections depend on the nature of the innovation process, we now assume that the vast majority of innovation events introduce cultural variants with low or medium adaptive values, i.e. variants with an adaptation value that does not exceed 0.8 in the current environmental state. This assumption renders adaptation through *d*e novo innovation less reliable and the waiting time for the introduction of a highly adaptive cultural variant increases.

#### 2.2.1 Repertoire size and structure.

As above, when environmental changes are frequent ($T_{\text{change}}<100$), vertical learning results in a fitness disadvantage (see Fig 1B, dark grey area). Compared to type I innovations, Fig 2A shows that the individual repertoires in populations engaging in vertical learning are smaller, even though forgetting rates have evolved to similar levels (see Fig B in S3). This reduction happens because when the innovation process is impaired, innovations that do yield a high adaptation value very quickly spread through the population leading to relatively homogeneous repertoires. Thus, individuals who have chosen to engage in social learning often observe variants that are already contained in their repertoires. By model assumption, those variants are not added to the repertoire again. Hence, social learning does not increase the size of the repertoire and individual repertoires are slightly less structured compared to the scenario with type I innovation where highly advantageous variants are overrepresented (compare Fig 3, first and second column).

Contrasting type II innovation populations with and without vertical learning, we see a similar dynamic as described in Sect 2.1.1. While the probabilities that at least one individual has information about the alternative environment at the point of an environmental change are very high when environmental change is rapid, the structure of the repertoires is different (see Fig 3, second column). Here, populations with vertical learning have a higher average repertoire size, containing more variants with low adaptation value compared to populations without vertical learning. This results, again, in a higher proportion and greater magnitude of sub-optimal choices (see Fig 2B, 2C and 2D) and lower fitness on average (see Fig 1B).

A less error-prone variant choice rule has the same effect as for type I innovation: the magnitude of sub-optimal choices is reduced, lowering the disadvantage of vertical learning (see Figs F and H in S4).

#### 2.2.2 Standing variation.

As for innovations of type I, for innovations of type II vertical learning provides an advantage when the environment becomes more stable, i.e. $T_{\text{change}}\geq 200$) (see Fig 1B). Fig 4B shows that the potential for adaptation through standing, unexpressed variation is higher for populations engaging in vertical learning and, at the same time, the structure of the individual cultural repertoires do not show substantial differences (see Figs 2A and 3). This implies that populations of non-vertical learners are more likely to rely on innovation. However, given the nature of the innovation process, a relatively large number of innovation events is needed to introduce a highly adaptive variant leading to the fitness differences (see Fig 1B).

For type II innovation there also exists a range of rates of environmental change in which the opposing effects of the two dynamics we have highlighted balance each other out (see light grey area Fig 1B). Both sub-optimal choices of expressed variants as well as the probability that at least one individual has information adapted to the alternative environment are similar in populations with and without vertical learning (see Figs D in S2 and 4B).

Again, similarly to type I innovation, under a less error-prone variant choice rule the advantage of vertical learning in relatively stable environments is further increased (see Fig F in S4).

### 2.3 Type III innovations

The nature of the innovation process can affect the cultural dynamic in complicated ways. In type III innovations, the vast majority of innovation events lead to the introduction of cultural variants with an adaptation value close to zero. This means that, compared to the type II innovations, the waiting times for the introduction of highly adaptive cultural variants are similar but all variants introduced until that point provide no adaptive value.

#### 2.3.1 Repertoire size and structure.

Fig 1C shows that, with this type of innovation, as with the others, vertical learning has a negative effect on adaptation in unstable environments, i.e. $T_{\text{change}}<100$. In this environmental range, again, populations with vertical learning have large individual repertoires (see Fig 2A). These repertoires are less structured compared to those in populations with innovations of type I, for example. They consist of highly adaptive variants but they also contain a vast number of variants with very low adaptation values (see Fig 3, third column).

Precisely this feature of the repertoire interacts with the evolution of forgetting rates. Under type I innovation, high forgetting rates lead to highly structured individual repertoires which reduce the severity of sub-optimal variant choices as compared to a scenario including the capacity for memory but excluding the ability to forget (for a detailed discussion of this feature of forgetting see [20]). However, under type III innovation the repertoires contain a large number of variants with adaptation values close to 0 (see Figs 3 and C in S2). These variants do not affect individual variant choice greatly because they are extremely rarely chosen and expressed. Consequently, forgetting rates evolve to lower levels (see Fig C in S3). In other words, under a type III innovation process it is much less important to structure the repertoires to yield a comparable proportion and magnitude of sub-optimal choices to type I and II innovations (see Fig 2B and 2C). With type III innovation, repertoires contain a few very highly adaptive variants which are frequently chosen, and a larger number of very poorly adaptive variants which are largely ignored.

As above, vertical learning does not provide an advantage in this very unstable environmental range compared to situations without vertical learning because the proportion and severity of sub-optimal choices are reduced in the absence of vertical learning (see Figs 2B, 2C, 2D and D in S2). The probabilities of preserving variants adapted to the alternative environment at the point of an environmental change are close to 1 for populations with and without vertical learning (see Fig 4C).

As for the other innovation types, a less error-prone variant choice rule reduces the magnitude of sub-optimal choices (see Fig M in S4), lowering the disadvantage of vertical learning (see Fig K in S4).

#### 2.3.2 Standing variation.

In environmental conditions with $T_{\text{change}}\geq 100$, Fig 1C shows that, with this type of innovation vertical learning has a positive effect on fitness.

The lower forgetting rates compared to type I and II innovations allow vertical learning to preserve unexpressed cultural variation with a higher probability (see Fig 4C). Populations of non-vertical learners, however, more often engage in innovation, which is relatively ineffective, leading to a striking advantage for vertical learning (see Fig 1) and a slightly higher maximum variant adaptation value for vertical learners (Fig 2D).

Here, a less error-prone variant choice rule only slightly further relaxes the selection pressure on forgetting (see Fig N in S4) and therefore the advantage of vertical learning in relatively stable environments remains almost unchanged (see Fig K in S4).

### 2.4 Further generalisation of the innovation process

In this section, we relax the assumption that a variant can only be adapted to one of the two considered environmental states. We assume that both states are sufficiently similar to allow the possibility that cultural variants developed in response to one environmental state also confer benefits in the alternative state.

First, we model a variant’s adaptation values in the two environmental states as correlated, random variables, each uniformly distributed on [0,1], with correlation coefficient ϱ=−0.9. Fig A in S5 illustrates the distribution of the variants’ adaptation values. Besides “specialist” variants showing a high adaptation value in one environmental state and a low adaptation value in the other, the assumption of negatively correlated adaptation values also allows for “generalist” variants which are characterized by similar but medium to low adaptation values in both states (see [24]). Furthermore, this modelling assumption implies that both environmental states are similar enough that variants invented in response to one environmental state can turn out to be better adapted in the other state. In very unstable environments, vertical learning becomes disadvantagous (see Fig B in S5) as the repertoires are larger than for the case of no vertical learning and — due to the way adaptation values are modelled — the repertoires are not as structured as, for example, in the case of type I innovation (see Figs C and D in S5). As the environment becomes more stable vertical learning has a very small advantage. High forgetting rates produce individual repertoires consisting of a single variant for most individuals. Consequently, vertical learning cannot contribute to the preservation of information adapted to the other environmental state — individuals aim to adapt to the current environmental conditions. The small fitness advantage arises from the fact that individuals do not need to learn or innovate at birth and the reduced number of innovations in particular slightly improves the fitness level of the population.

Second, to study the most extreme situation, we impose no restriction on the innovation process and assume that the adaptation value of new variants is drawn uniformly from [0,1] in both states. Now, vertical learning has negligible effects across most environmental regimes, with a small advantage only in very stable environments (see Fig F in S5). High forgetting rates evolve (see Fig I in S5), reducing repertoires to less than two variants on average (see Fig H in S5). Consequently, populations typically fix a single “Über-variant” that is well adapted to both states - a phenomenon previously described elsewhere [24]. Vertical learning does not increase the probability that such a broadly adaptive variant exists (Fig J in S5 shows that a large fraction of the population carries a variant with an adaptation value ≤0.95 in both states). The slight advantage of vertical learning in stable environments stems from parental transmission enabling highly adaptive variants to spread rapidly without the need for further innovation.

## 3 Discussion

The model presented here allows for the acquisition of cultural variants through learning or innovation, the generation of individual cultural repertoires through memory and forgetting, and their persistence across generations through vertical learning. The model explores the benefit of vertical learning as the environment changes and when innovation is easier or more difficult. Previous analyses of the evolution of vertical learning have shown that vertical learning is favoured when the environment is very stable and information gained and used by this generation is likely to be useful to the next (e.g., [6,18,23]). Here we have shown that with the capacity for memory, and assuming that learning can occur multiple times in an individual’s lifetime, the benefit of vertical learning critically depends on the interplay between the size of individual repertoires and the duration of environmental stasis. Overall, we find that vertical learning is most advantageous when environments vary at intermediate rates and innovation is particularly difficult.

In the absence of vertical learning, cultural adaptation after an environmental change requires either rapid innovation or social learning from other individuals who have retained or discovered adaptive cultural variants. Additionally, naive individuals have to engage in either social learning or innovation when vertical learning is absent. Our results show that vertical learning provides a slight benefit to the offspring in highly stable environments, when their parent’s knowledge is up-to-date and can be copied with low risk. This effect is most obvious for scenarios in which a single cultural variant can be highly adapted to both environmental states (see S5 Sect) and the generation of individual repertoires is obsolete. The major benefit of vertical learning in our model comes from its ability to allow unexpressed cultural variation suitable for alternative environmental states to persist and be carried across generations. This means that at intermediate rates of environmental change, individuals need not rely on innovation in order to adjust to their new environments, rather, they can rely on the cultural variants contained within their repertoires. The benefit of this is especially visible when innovation is most difficult (here, we referred to this case as ‘type III innovation’). Reliance on innovation in this case would mean that individuals must wait a considerable time before they discover a suitable cultural variant. This ‘waiting time’ can be eradicated or greatly shortened by vertical learning, which maintains larger repertoires. When the number of individuals who have repertoires containing useful cultural variation decreases, social learning plays a crucial role—even where vertical learning is possible. It is sufficient that one or two individuals in the population maintain useful variation, this can be spread rapidly through the population via social learning. We show that the preservation of variants depends critically on the evolution of forgetting, which is not solely dominated by the process of innovation: An almost deterministic variant choice rule can relax selection pressure on forgetting, thereby increasing the probability of preserving variants adapted to the alternative environmental state. As a consequence, the advantage of vertical learning is increased.

The preservation of a large number of cultural variants over long periods of time is a double-edged sword. In intermediate and stable environments, this is beneficial. In rapidly changing environments, however, these preserved repertoires become increasingly large and less structured compared to smaller repertoires beginning to interfere with an individual’s ability to choose cultural variants effectively (see also, [20]). Therefore when the environment changes rapidly and cultural repertoires become large and less structured, vertical learning is detrimental. However, we show that this disadvantage can be mitigated by a less error-prone variant choice rule (see S4 Sect).

In our model, different innovation types lead to nuanced effects. We contrasted three types of innovation: one that produces many good innovations, one that produces many innovations of moderate value and occasional innovations of very high value, and one that produces many poor innovations and occasional innovations of very high value. In the latter cases, the probability of producing an extremely good variant was the same. We found that producing many moderate innovations was more detrimental than producing many poor innovations. Poor innovations were easily recognised by individuals and rarely, if ever, chosen from the repertoire. When many moderate innovations were produced, individuals chose these at a much higher rate. This led to the counter-intuitive result that if innovation is to fail, it is best that it fails completely. We note that this result interacts critically with our assumptions about how individuals choose variants from their repertoires.

These findings are largely in concordance with the results of previous models which also suggest a role for vertical learning in relatively stable environmental conditions. A noteworthy outcome of our model is that, unlike in some previous work (e.g., [18]), the benefit of vertical learning does not continue to increase, nor does it stabilise at a high value, as the environment becomes increasingly stable and unexpressed variants are erased from the repertoire before the next change occurs due to high forgetting rates. Based on our model, for each case we have considered, there appears to be some value of environmental stability for which the fitness levels of populations that engage in vertical learning and those that do not, equalise, or become very similar. The point at which this happens is different for different types of innovation.

We note that our model, as with the model of Ammar et al. [20], has made several simplifying assumptions about the ways in which memory, forgetting, and decision making proceed. We have relaxed several assumptions of previous models on similar topics, for example by allowing several learning events per lifetime, by allowing individuals to engage in vertical learning, social learning, and innovation, and by allowing individuals to possess sophisticated and dynamic memories. Although this brings our model in closer alignment with previous work on, for example, enculturation, it would certainly be possible to refine and improve our representation of cognitive processes involved in learning and memory, perhaps with implications for our results.

## Supporting information

S1 File S1–S5 The supplementary material provides more information on some of the model’s assumptions (S1) and explanations of the outcomes (S2 and S3). It further contains additional analyses (S4–S5).
**S1 Section: Age and lifespan distribution**
– Fig A in S1: Distribution of ages and deaths**S2 Section: Distribution and accumulation and choice of cultural variants**
– Fig A in S2: Repertoire composition for type I innovation– Fig B in S2: Repertoire composition for type II innovation– Fig C in S2: Repertoire composition for type III innovation– Fig D in S2: Repertoire size and sub-optimal choices– Fig E in S2: Repertoire size and average benefit**S3 Section: Fitness levels and the evolution of social learning and forgetting**
– Fig A in S3: Fitness, social learning, and forgetting for type I innovation– Fig B in S3: Fitness, social learning, and forgetting for type II innovation– Fig C in S3: Fitness, social learning, and forgetting for type III innovation**S4 Section: Alternative variant choice rule: Softmax, *τ* = 0.1****Type I innovation with SoftMax**
– Fig A in S4: Fitness difference– Fig B in S4: Repertoire composition– Fig C in S4: Repertoire size and sub-optimal choices– Fig D in S4: Fitness, social learning, and forgetting– Fig E in S4: Probability of preservation of variants**Type II innovation with SoftMax**
– Fig F in S4: Fitness difference– Fig G in S4: Repertoire composition– Fig H in S4: Repertoire size and sub-optimal choices– Fig I in S4: Fitness, social learning, and forgetting– Fig J in S4: Probability of preservation of variants**Type III innovation with SoftMax**
– Fig K in S4: Fitness difference– Fig L in S4: Repertoire composition– Fig M in S4: Repertoire size and sub-optimal choices– Fig N in S4: Fitness, social learning, and forgetting– Fig O in S4: Probability of preservation of variants**S5 Section: Alternative innovation processes****Innovation of correlated adaptation values for both environmental states**
– Fig A in S5: Distribution of adaptation values– Fig B in S5: Fitness difference– Fig C in S5: Repertoire composition– Fig D in S5: Repertoire size and sub-optimal choices– Fig E in S5: Fitness, social learning, and forgetting**Innovation of uniformly distributed adaptation values for both environmental states**
– Fig F in S5: Fitness difference– Fig G in S5: Repertoire composition– Fig H in S5: Repertoire size and sub-optimal choices– Fig I in S5: Fitness, social learning, and forgetting– Fig J in S5: Frequency of variants maximally adapted to both environments
(PDF)
