# Supplementary material for: Memory, innovation and vertical learning
Source: PLoS Comput Biol. 2025 Dec 5;21(12):e1013785. doi: 10.1371/journal.pcbi.1013785 (PMC12697990; doi:10.1371/journal.pcbi.1013785)
Supplement: S1 File — S1–S5 The supplementary material provides more information on some of the model’s assumptions (S1) and explanations of the outcomes (S2 and S3). It further contains additional analyses (S4–S5). S1 Section: Age and lifespan distribution – Fig A in S1: Distribution of ages and deathsS2 Section: Distribution and accumulation and choice of cultural variants – Fig A in S2: Repertoire composition for type I innovation– Fig B in S2: Repertoire composition for type II innovation– Fig C in S2: Repertoire composition for type III innovation– Fig D in S2: Repertoire size and sub-optimal choices– Fig E in S2: Repertoire size and average benefitS3 Section: Fitness levels and the evolution of social learning and forgetting – Fig A in S3: Fitness, social learning, and forgetting for type I innovation– Fig B in S3: Fitness, social learning, and forgetting for type II innovation– Fig C in S3: Fitness, social learning, and forgetting for type III innovationS4 Section: Alternative variant choice rule: Softmax, τ = 0.1Type I innovation with SoftMax – Fig A in S4: Fitness difference– Fig B in S4: Repertoire composition– Fig C in S4: Repertoire size and sub-optimal choices– Fig D in S4: Fitness, social learning, and forgetting– Fig E in S4: Probability of preservation of variantsType II innovation with SoftMax – Fig F in S4: Fitness difference– Fig G in S4: Repertoire composition– Fig H in S4: Repertoire size and sub-optimal choices– Fig I in S4: Fitness, social learning, and forgetting– Fig J in S4: Probability of preservation of variantsType III innovation with SoftMax – Fig K in S4: Fitness difference– Fig L in S4: Repertoire composition– Fig M in S4: Repertoire size and sub-optimal choices– Fig N in S4: Fitness, social learning, and forgetting– Fig O in S4: Probability of preservation of variantsS5 Section: Alternative innovation processesInnovation of correlated adaptation values for both environmental states – Fig A in S5: Distribution of adaptation values– Fig B in S5: Fitness dif [file pcbi.1013785.s001.pdf]

Supplementary material to Memory, innovation and vertical learning

Madeleine Ammar[1], Laurel Fogarty[1], and Anne Kandler[1]

[1]TICE lab, Department of Human Behavior, Ecology, and Culture, Max Planck Institute for Evolutionary Anthropology, Deutscher Platz 6, 04103 Leipzig

December 1, 2025

Corresponding Author: Madeleine Ammar

Email: madeleine.ammar@eva.mpg.de

This PDF includes:

- Supporting text
- Figure A in S1 text
- Figures A-E in S2 text
- Figures A-C in S3 text
- Figures A-O in S4 text
- Figures A-J in S5 text

S1 Age and lifespan distribution

In Section 4.4 in the main text, we explain that death is an age-dependent process. Individuals of higher age have a higher probability of dying than young individuals. This modification of the classic Moran model, which imposes a random death process on individuals, curtails the chance that some individuals may happen to live much longer than others. Both populations exhibit similar average lifespans, namely 198.64 in populations with an age-dependent death process, and 198.67 in populations with a random death process. This approximates the theoretical expectation of an average lifespan of 200 in a Moran model, which we would achieve with a higher number of simulations. Fig A in S1 shows that an age-dependent death process narrows the age distribution (left column), while the distribution of lifespans is shifted towards higher ages (right column).

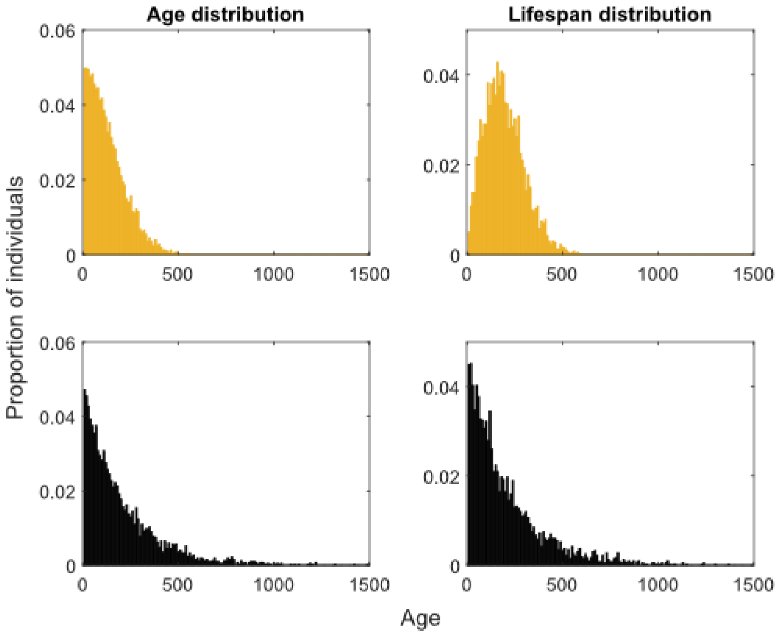

Figure A: Distribution of ages (left column) and lifespans (right column) in populations with an age-dependent death process (upper row, yellow) and populations with a random death process (lower row, black). Distributions are recovered from 20 populations of each type.

A

S2 Distribution and accumulation of cultural variants and its effect on variant choice

In the main text we describe how events of social learning and innovation together with the storage and forgetting of variants result in the emergence of individuals' cultural repertoires. Repertoires are largely influenced by rates of forgetting and social learning propensities that evolve in response to environmental variability (see Figs A-C in S2). In Fig D in S2, we depict how processes of learning, memory, and forgetting are reflected in repertoire size and two aspects of variant choice from these repertoires: 1) the frequency of choosing a sub-optimal variant from the repertoire, and 2) the magnitude of sub-optimal variant choices. Of focal interest is the difference between populations with and without vertical transmission in their accumulation of variants and its effects on the efficiency of variant choice. In Fig E in S2, we show the effect of repertoire's sizes on its expected benefit. Applying the variant choice rule described in Eq. (1) to repertoires of increasing size, we obtain decreasing average benefit levels.

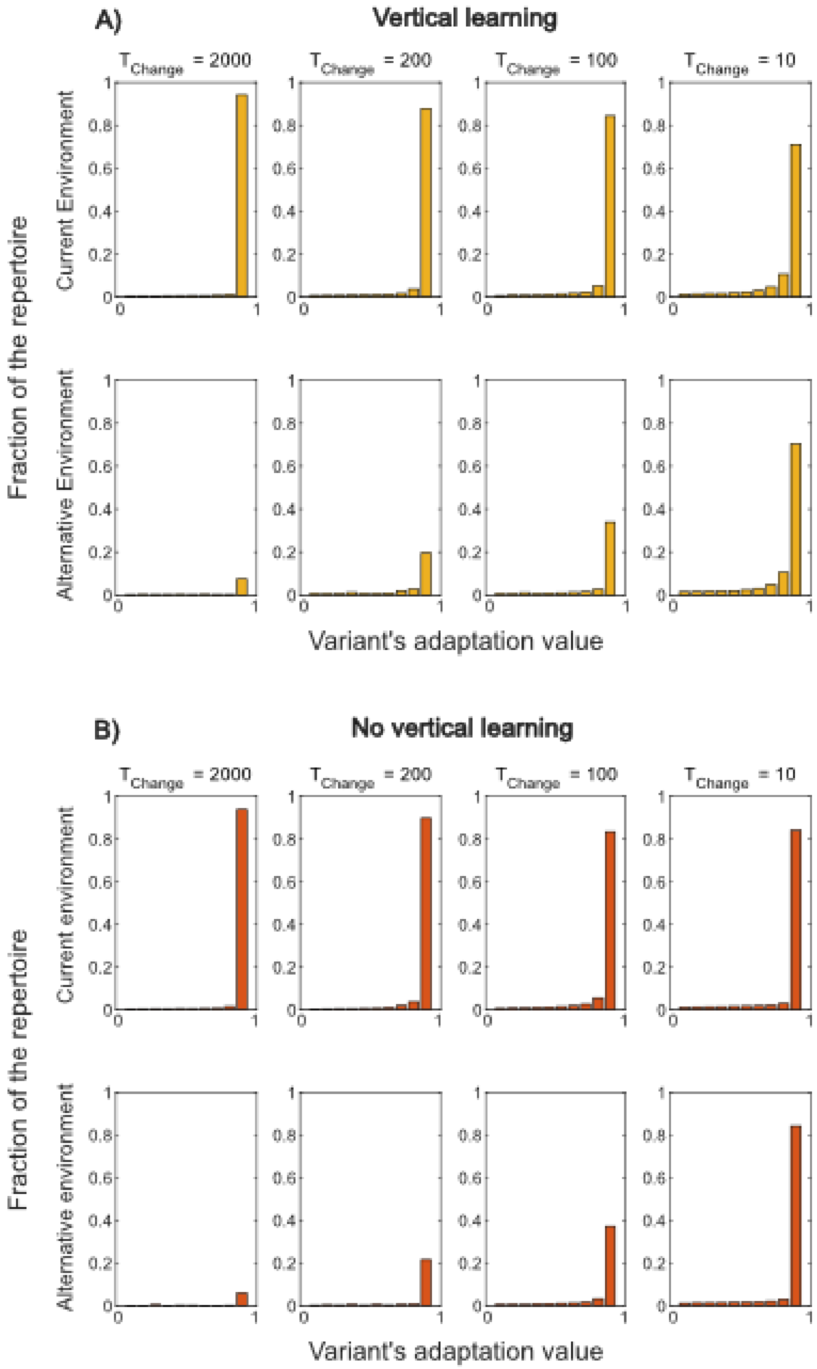

Figure A: Repertoire composition at different T_{change} values (columns) with vertical learning (A) and no vertical learning (B). Each bar shows the fraction of cultural variants that fall within a given adaptation interval, ranging from 0 (lowest adaptation value, left) to 1 (highest adaptation value, right). Fractions are calculated for single repertoires and further averaged over all simulations. The top row represents cultural variants adapted to the current environment, the bottom row represents variants adapted to the alternative environment. Results correspond to type I innovations.

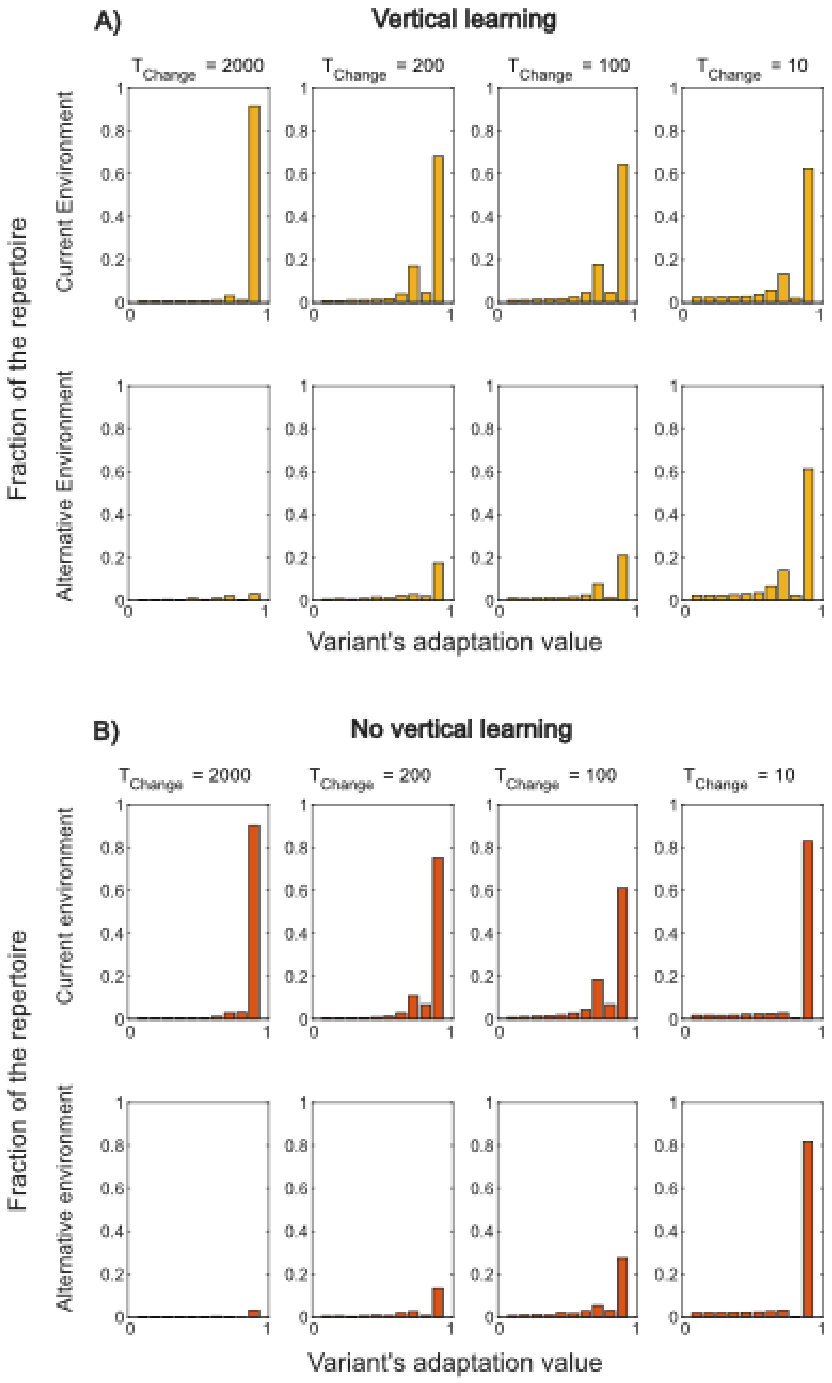

Figure B: Repertoire composition at different T_{change} values (columns) with vertical learning (A) and no vertical learning (B). Each bar shows the fraction of cultural variants that fall within a given adaptation interval, ranging from 0 (lowest adaptation value, left) to 1 (highest adaptation value, right). Fractions are calculated for single repertoires and further averaged over all simulations. The top row represents cultural variants adapted to the current environment, the bottom row represents variants adapted to the alternative environment. Results correspond to type II innovations.

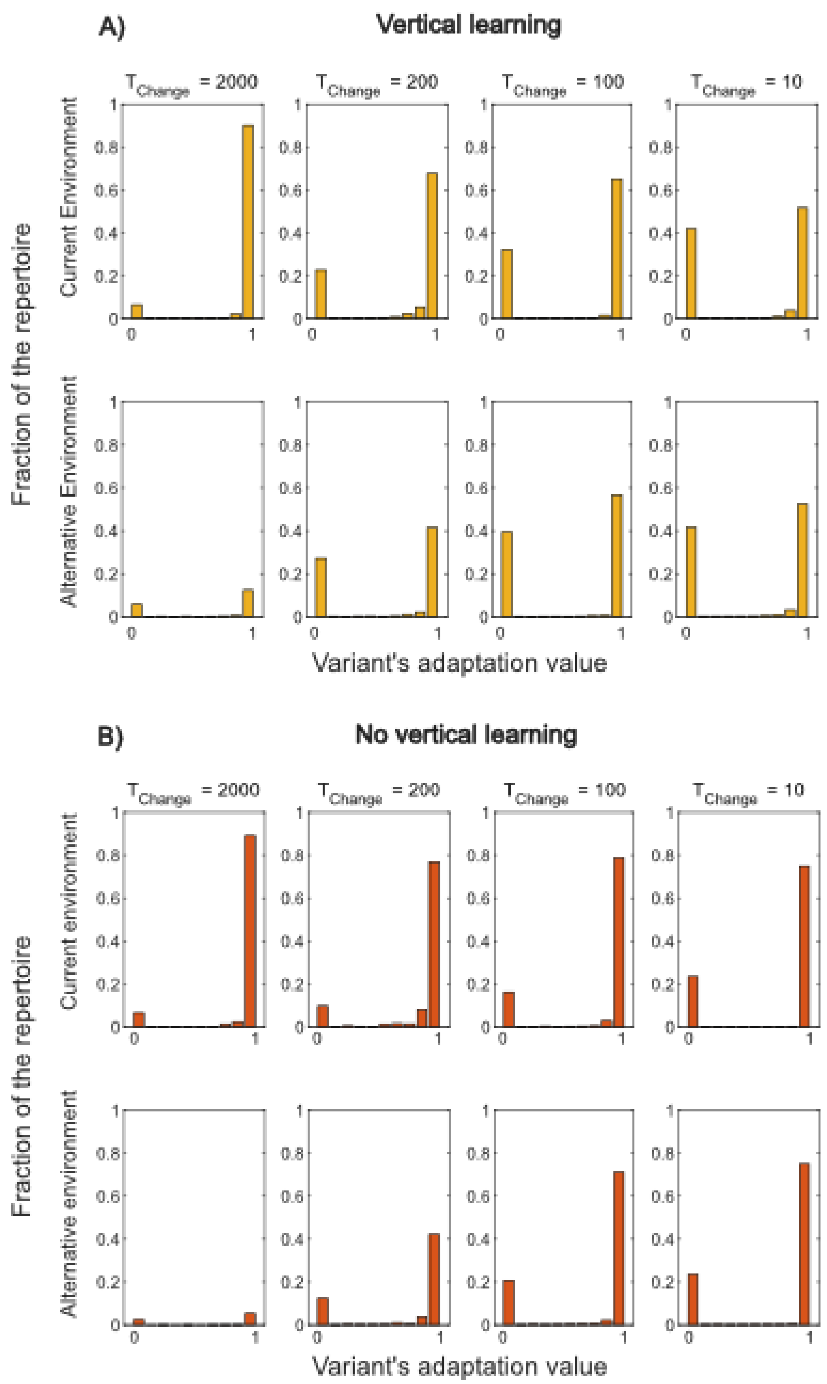

Figure C: Repertoire composition at different T_{change} values (columns) with vertical learning (A) and no vertical learning (B). Each bar shows the fraction of cultural variants that fall within a given adaptation interval, ranging from 0 (lowest adaptation value, left) to 1 (highest adaptation value, right). Fractions are calculated for single repertoires and further averaged over all simulations. The top row represents cultural variants adapted to the current environment, the bottom row represents variants adapted to the alternative environment. Results correspond to type III innovations.

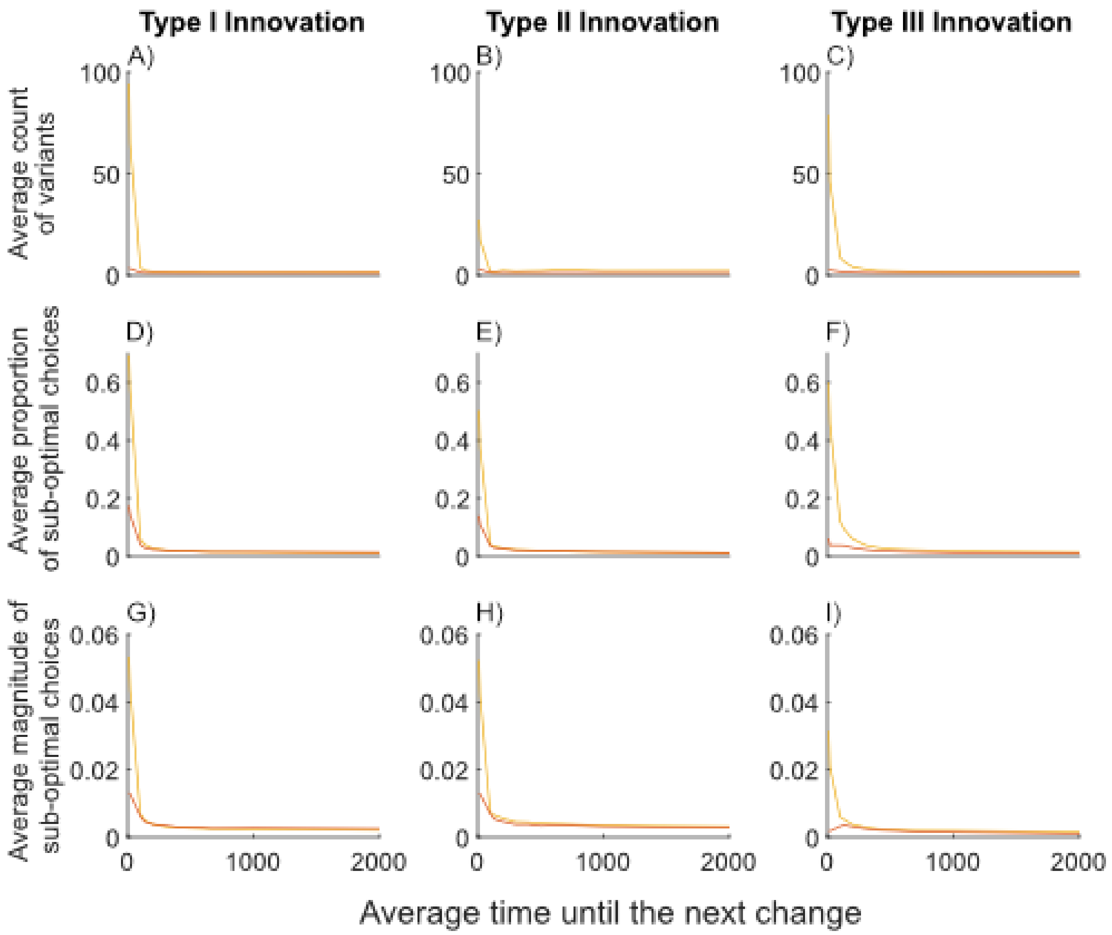

Figure D: Relationship between environmental variability and the average count of variants (top row), the average proportion of sub-optimal variant choices per individual's lifetime (middle row), and the average magnitude of sub-optimal choices (bottom row). Values in the top row are calculated as the average variant count per individual in the last generation (i.e. 200 time steps) of a simulation. Values in the middle row are calculated as the age-normalized proportion of sub-optimal variant choice of individuals living in the last generation of a simulation. Values in the bottom row are calculated as the age-normalized average distance between individual's sub-optimal variant choice and the best variant in their repertoire. Population averages are further averaged over all simulations. Yellow lines correspond to simulations including vertical learning, red lines correspond to simulations without vertical learning. Columns represent the different innovation types.

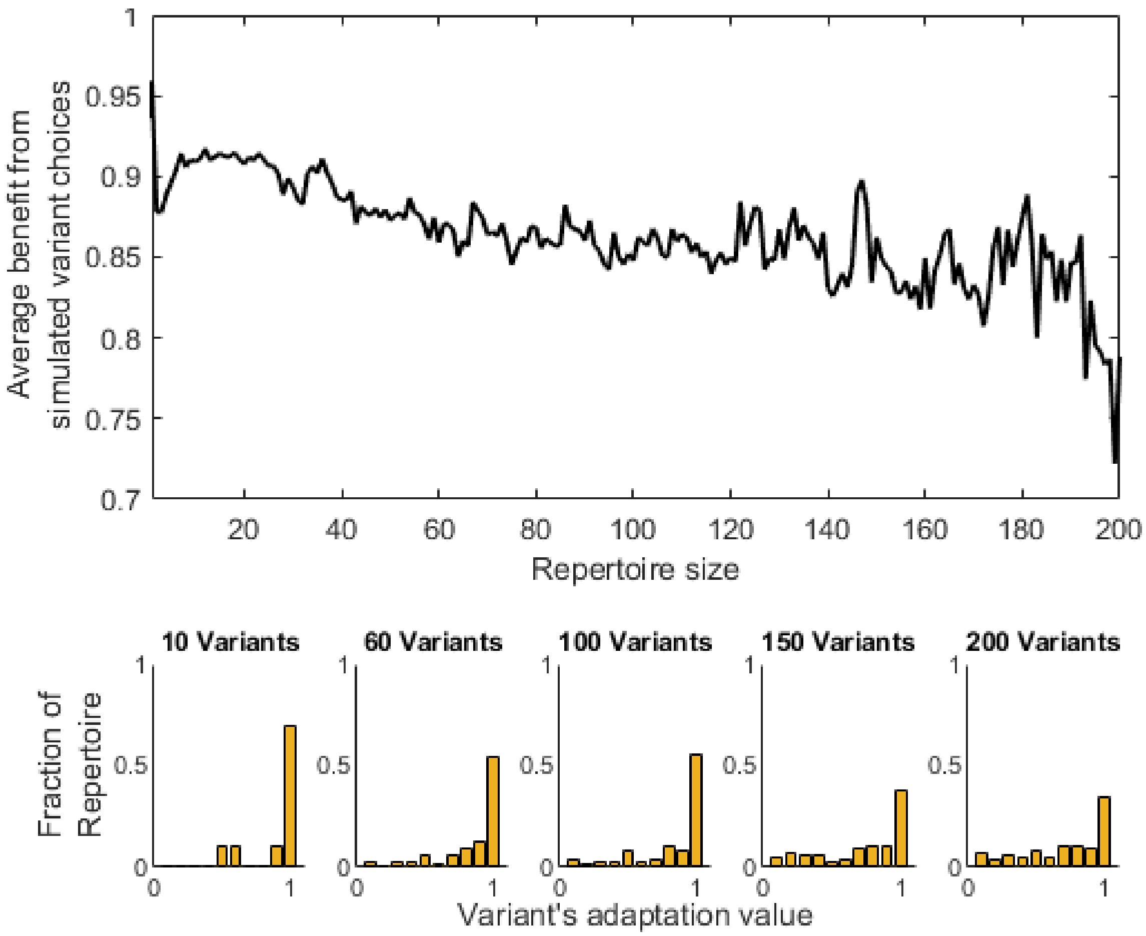

Figure E: Relationship between repertoire's size and repertoire's average benefit level. For each individual, we calculate the average benefit of their repertoire by choosing 100 variants from it according to decision rule (1) from the main text and averaging over their adaptation values. The top panel plots the average benefit per repertoire size against repertoire size. Values are calculated for populations with vertical learning and type I innovation at $T_{\text{change}} = 100$. The bottom row shows exemplary compositions of single repertoires consisting of 10, 60, 100, 150 or 200 cultural variants.

A

S3 Fitness levels and the evolution of social learning and forgetting

Populations with and without vertical learning live in environments of different environmental variability. Individual's expression of a cultural variant represents the individual's fit to the environment and consequently forms the basis for our calculation of the population's fitness. This eventually feeds back in the evolution of social learning propensities and forgetting rates. We compare the corresponding trajectories for populations with and without vertical learning. Comparisons are separated for the three innovation regimes.

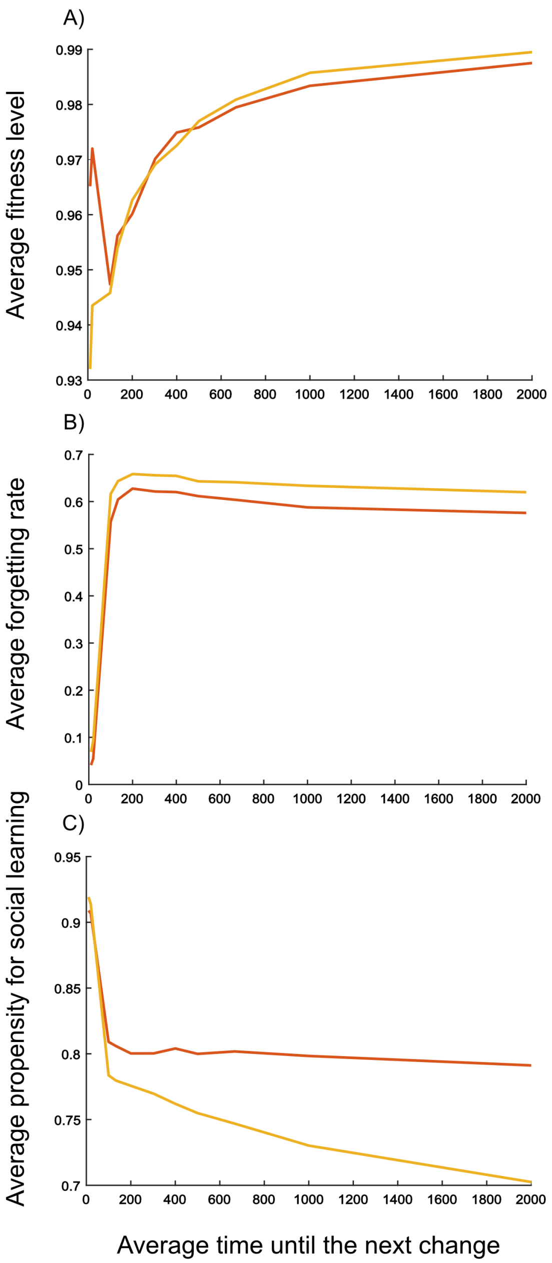

Figure A: Relationship between environmental variability and average (A) population level fitness, (B) rate of forgetting, (C) social learning propensity. Values are calculated as the average values for all individuals in the last generation (i.e. 200 time steps) of a simulation (N = 200). Population averages are further averaged over all simulations. Yellow lines correspond to simulations including vertical learning, red lines correspond to simulations without vertical learning. All results correspond to type I innovations.

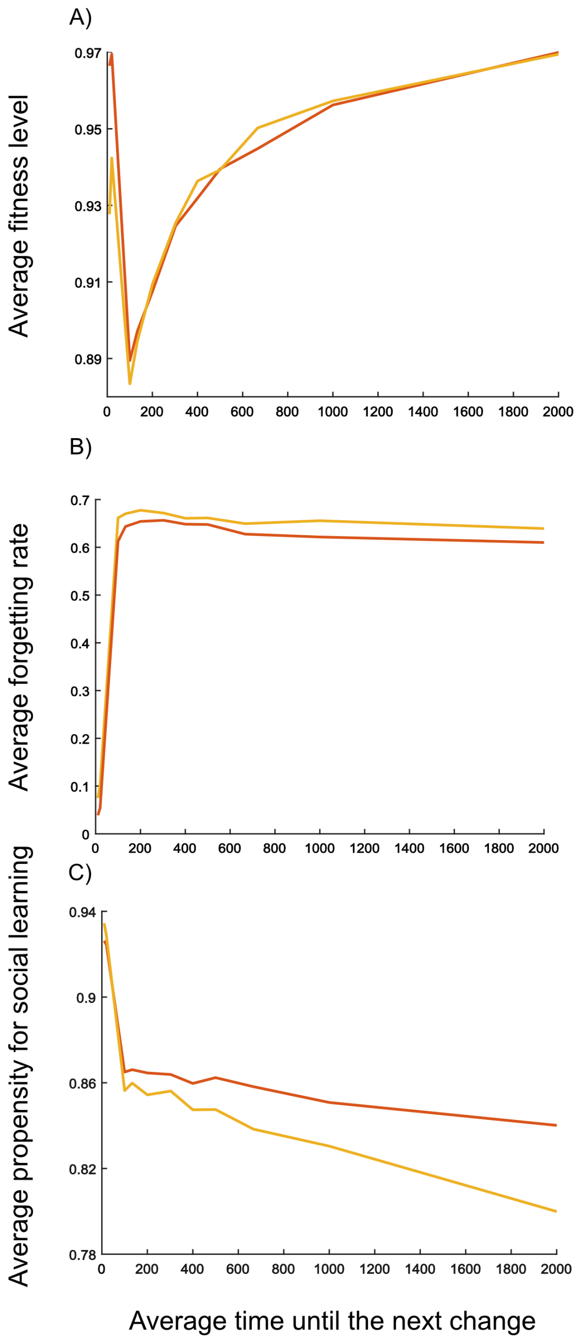

Figure B: Relationship between environmental variability and average (A) population level fitness, (B) rate of forgetting, (C) social learning propensity. Values are calculated as the average values for all individuals in the last generation (i.e. 200 time steps) of a simulation (N = 200). Population averages are further averaged over all simulations. Yellow lines correspond to simulations including vertical learning, red lines correspond to simulations without vertical learning. All results correspond to type II innovations.

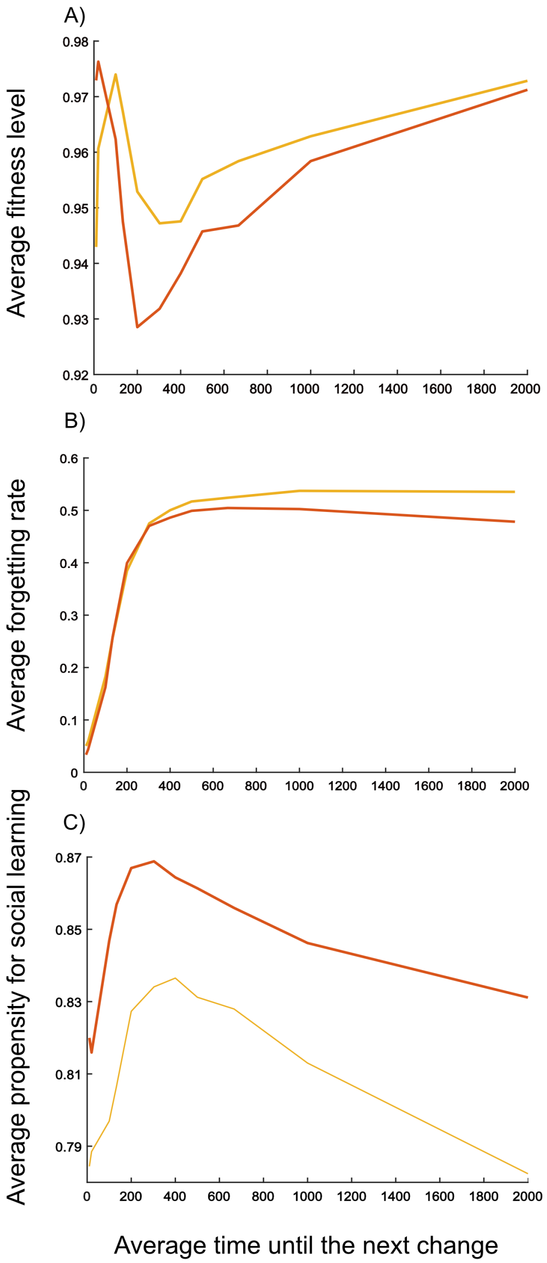

Figure C: Relationship between environmental variability and average (A) population level fitness, (B) rate of forgetting, (C) social learning propensity. Values are calculated as the average values for all individuals in the last generation (i.e. 200 time steps) of a simulation (N = 200). Population averages are further averaged over all simulations. Yellow lines correspond to simulations including vertical learning, red lines correspond to simulations without vertical learning. All results correspond to type III innovations.

A

S4 Alternative variant choice rule: Softmax, τ =0.1

In Eq. (1) we describe the variant choice rule that individuals employ when choosing which variant to express from their cultural repertoires. Following this rule, an individual is more likely to choose variants with a high adaptation value from its repertoire. However, as cultural repertoires increase in size and become comparatively less structured, individuals are prone to more errors in their decision making (see Figs 2 and D in S2).

Here, we test an alternative variant choice rule, softmax, that is particularly prominent in the field of reinforcement learning. This rule is often applied to study the effect of balancing the exploration and exploitation of behaviors through manipulation of individual's sensitivity to the benefit of a behavior. In other words, if individuals show no sensitivity to the benefit of a behavior, they will explore behaviors at random. If, on the other hand, individuals show great sensitivity they will choose highly beneficial behaviors quite often. We replace Eq. (1) with the softmax rule:

$$p_i^j(k) = \frac{exp(a_i(k)/\tau)}{\sum\limits_{s \in M^j} exp(a_s(k)/\tau)}, \tag{S4}$$

where τ controls the sensitivity to the benefit of variants in individual's cultural repertoire. Here, we show the results for low values of τ, i.e. when individuals exploit their highly beneficial variants almost deterministically. We focus on the effect of this new rule on the benefit of vertical learning.

Figs A, F, and K in S4 show that the benefit of vertical learning matches the qualitative result we see under the probabilistic variant choice rule (see Fig 1): Vertical learning is deleterious in highly variable environments and most beneficial when environments change at an intermediate frequency. However, we see a relative decrease in the disadvantage of vertical learning in variable environments and a relative increase in the advantage of vertical learning in more stable environments. The former can be explained by the almost deterministic exploitation of highly beneficial variants, which diminishes the magnitude of sub-optimal choices (see Figs C, H, and M in S4), and consequently reduces the deleterious effect of large and less structured repertoires in variable environments. A less error-prone variant choice rule relaxes selection pressure on forgetting and forgetting evolves to lower rates (see Figs D, I, and N in S4). As a consequence, individuals exhibit larger repertoires for smaller T_{Change} (see Figs C, H, and M in S4) that allow for a higher probability of preserving variants adapted to the alternative environment (see Figs E, J, and O) and thus increase the benefit of vertical learning in this range.

Surprisingly, Figs E, J, and O show that although the probability of preserving variants is increased in very stable environments (for comparison, see Fig 4), the benefit of vertical learning in this range is almost negligible (for comparison, see Fig 1). In this range, the relaxed selection pressure on forgetting results in a slightly higher frequency of sub-optimal choices than under a probabilistic variant choice (see Figs C, H, and M in S4). This disadvantage of vertical learning can not be compensated by the increased probability of preserving variants until the next changes occurs, since optimal performance in the current environment is more important than preservation of variants adapted to the alternative state when environmental changes are rare.

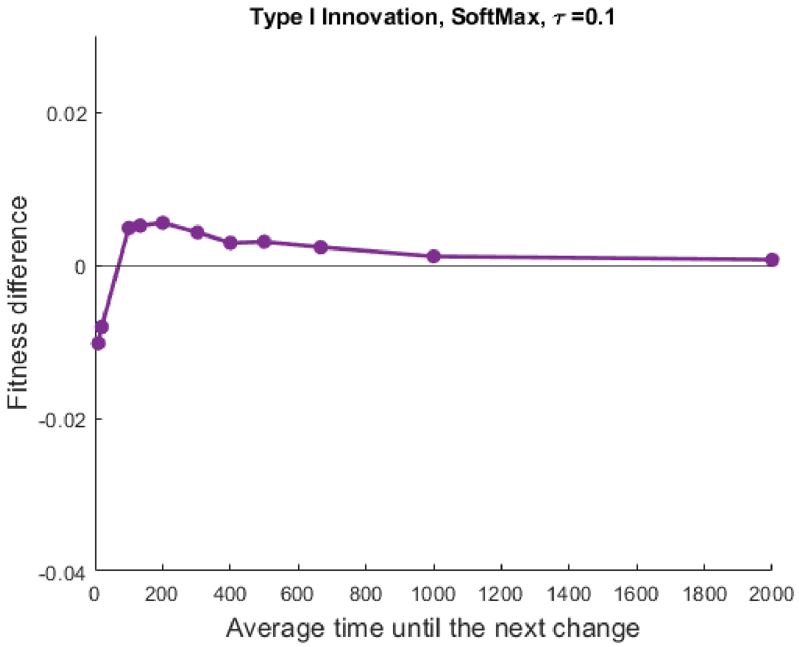

Figure A: Relationship between environmental variability and average fitness difference between populations with and without vertical learning. Values are calculated as the average fitness value received as the average expression value of variants by all individuals in the last generation (i.e. 200 time steps) of a simulation. Fitness values of populations without vertical learning are subtracted from populations exhibiting vertical learning. Population averages are further averaged over all simulations. Results correspond to type I innovations with a softmax rule.

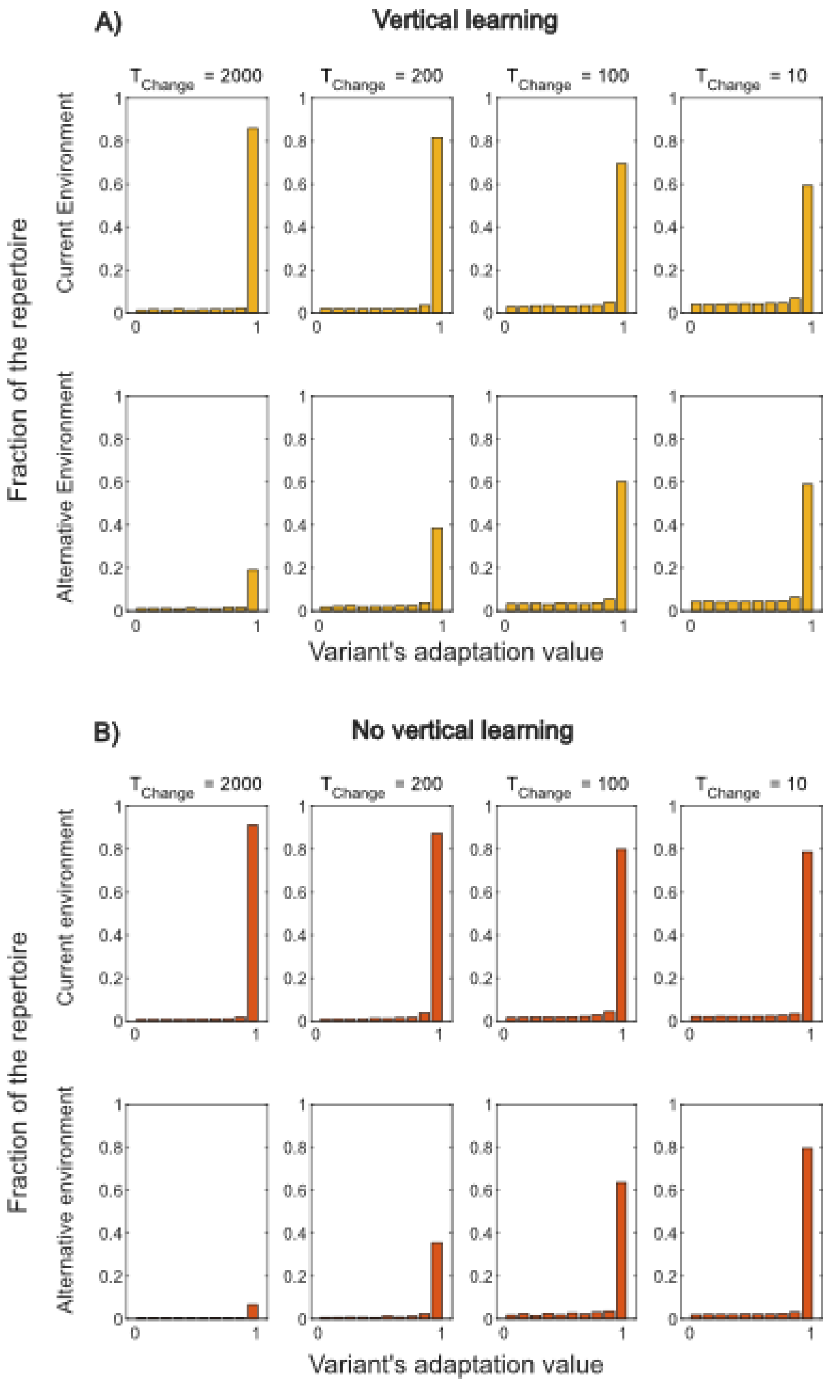

Figure B: Repertoire composition at different T_{change} values (columns) with vertical learning (A) and no vertical learning (B). Each bar shows the fraction of cultural variants that fall within a given adaptation interval, ranging from 0 (lowest adaptation value, left) to 1 (highest adaptation value, right). Fractions are calculated for single repertoires and further averaged over all simulations. The top row represents cultural variants adapted to the current environment, the bottom row represents variants adapted to the alternative environment. Results correspond to type I innovations with a softmax rule.

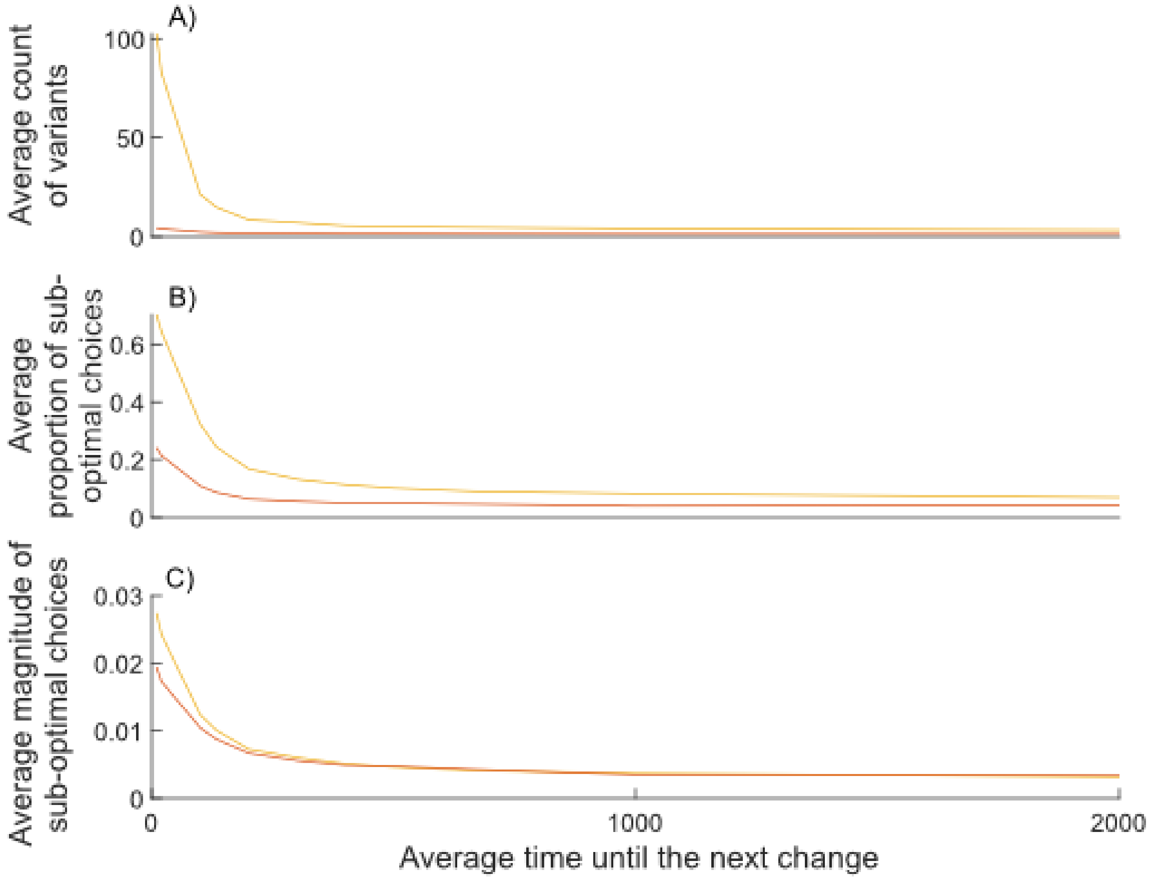

Figure C: Repertoire size and features sub-optimal variant choice at different T_{change} values (x-axis). A) Average count of variants: Values are calculated as the average variant count per individual in the last generation (i.e. 200 time steps) of a simulation. B) Average proportion of individual's sub-optimal variant: Values are calculated as the age-normalized proportion of sub-optimal variant choices of individuals living in the last generation of a simulation. C) Average magnitude of sub-optimal choice: Values are calculated as the age-normalized average distance between individual's sub-optimal variant choice and the best variant in their repertoire. Averages are calculated for individuals living in the last generation of a simulation. Population averages are further averaged over all simulations. Yellow line corresponds to the population with vertical learning, red line corresponds to the population without vertical learning. Results correspond to type I innovations with a softmax rule.

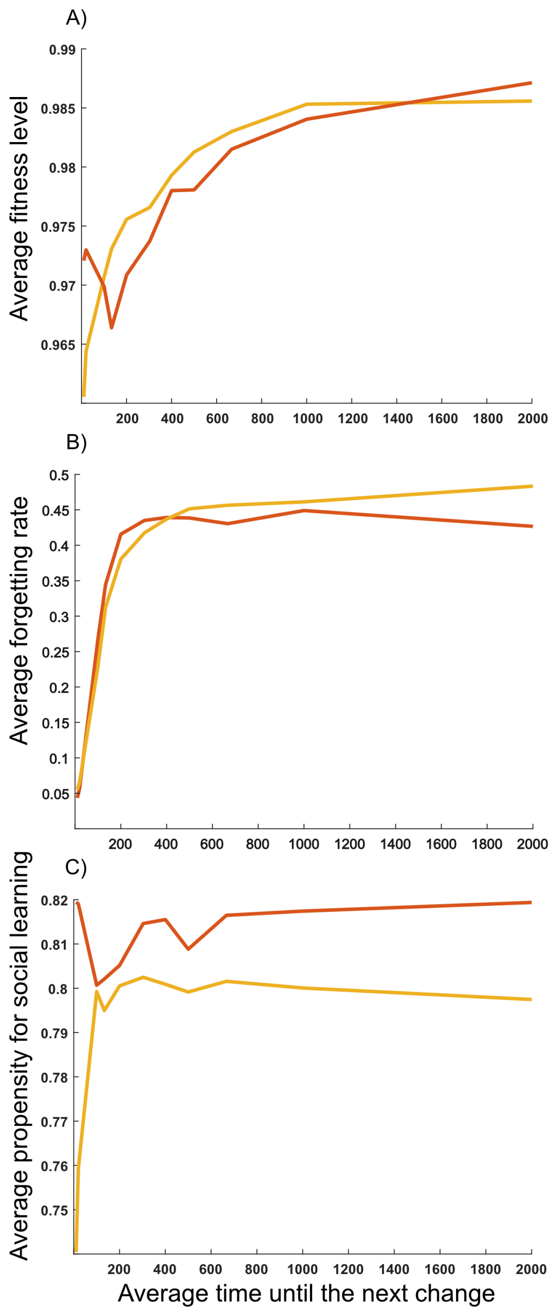

Figure D: Relationship between environmental variability and average (A) population level fitness, (B) rate of forgetting, (C) social learning propensity. Values are calculated as the average values for all individuals in the last generation (i.e. 200 time steps) of a simulation (N = 200). Population averages are further averaged over all simulations. Yellow lines correspond to simulations including vertical learning, red lines correspond to simulations without vertical learning. Results correspond to type I innovations with a softmax rule.

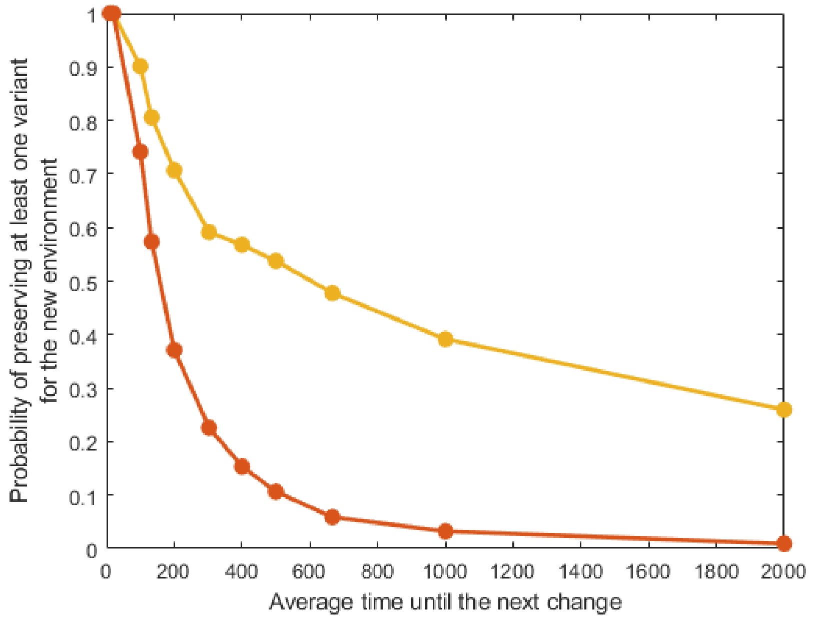

Figure E: Relationship between environmental variability and the probability of preserving at least one variant adapted to the 'alternative' environment until the next change occurs. Simulations are run until the burn-in phase and stopped once the first change occurs (before learning takes place). For each simulation it is recorded whether the population consists of at least one individual that knows about a variant adapted to the 'alternative' environment. Values are averaged over all simulations. Yellow lines correspond to simulations with vertical learning, red lines correspond to simulations without vertical learning. Results correspond to type I innovations with a softmax rule.

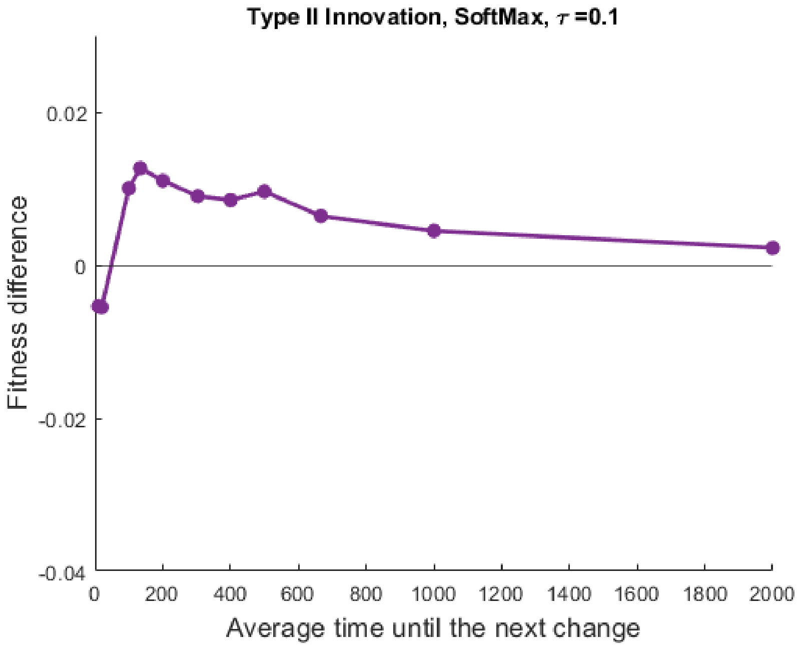

Figure F: Relationship between environmental variability and average fitness difference between populations with and without vertical learning. Values are calculated as the average fitness value received as the average expression value of variants by all individuals in the last generation (i.e. 200 time steps) of a simulation. Fitness values of populations without vertical learning are subtracted from populations exhibiting vertical learning. Population averages are further averaged over all simulations. Results correspond to type II innovations with a softmax rule.

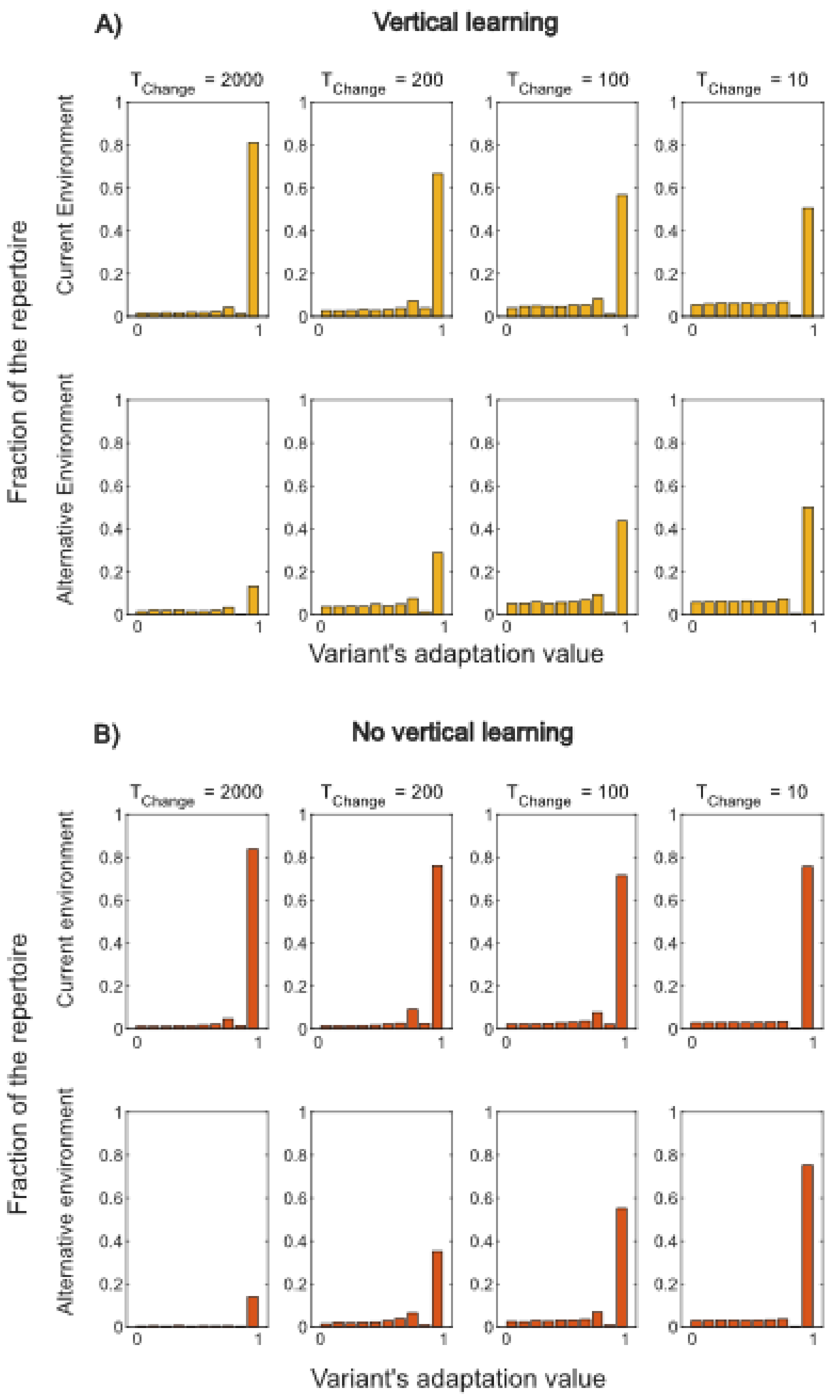

Figure G: Repertoire composition at different T_{change} values (columns) with vertical learning (A) and no vertical learning (B). Each bar shows the fraction of cultural variants that fall within a given adaptation interval, ranging from 0 (lowest adaptation value, left) to 1 (highest adaptation value, right). Fractions are calculated for single repertoires and further averaged over all simulations. The top row represents cultural variants adapted to the current environment, the bottom row represents variants adapted to the alternative environment. Results correspond to type II innovations with a softmax rule.

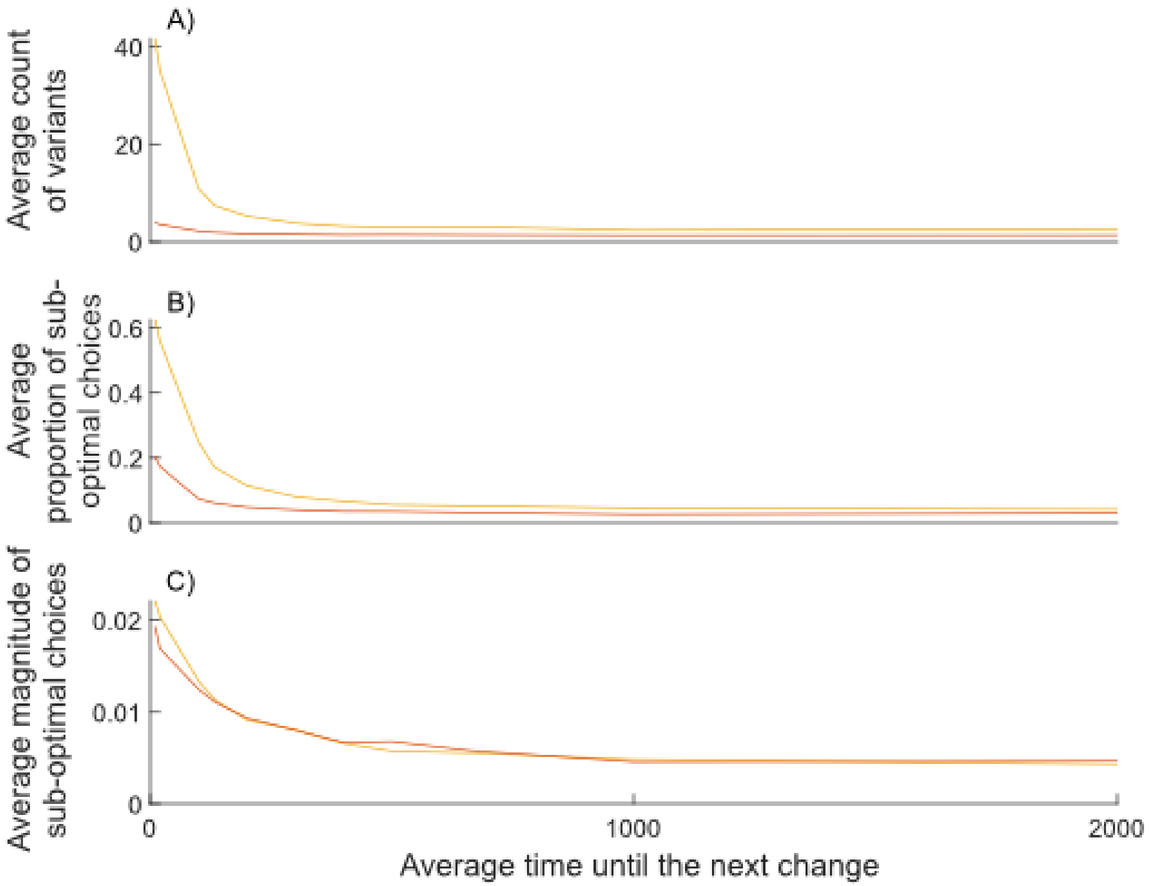

Figure H: Repertoire size and features sub-optimal variant choice at different T_{change} values (x-axis). A) Average count of variants: Values are calculated as the average variant count per individual in the last generation (i.e. 200 time steps) of a simulation. B) Average proportion of individual's sub-optimal variant: Values are calculated as the age-normalized proportion of sub-optimal variant choices of individuals living in the last generation of a simulation. C) Average magnitude of sub-optimal choice: Values are calculated as the age-normalized average distance between individual's sub-optimal variant choice and the best variant in their repertoire. Averages are calculated for individuals living in the last generation of a simulation. Population averages are further averaged over all simulations. Yellow line corresponds to the population with vertical learning, red line corresponds to the population without vertical learning. Results correspond to type II innovations with a softmax rule.

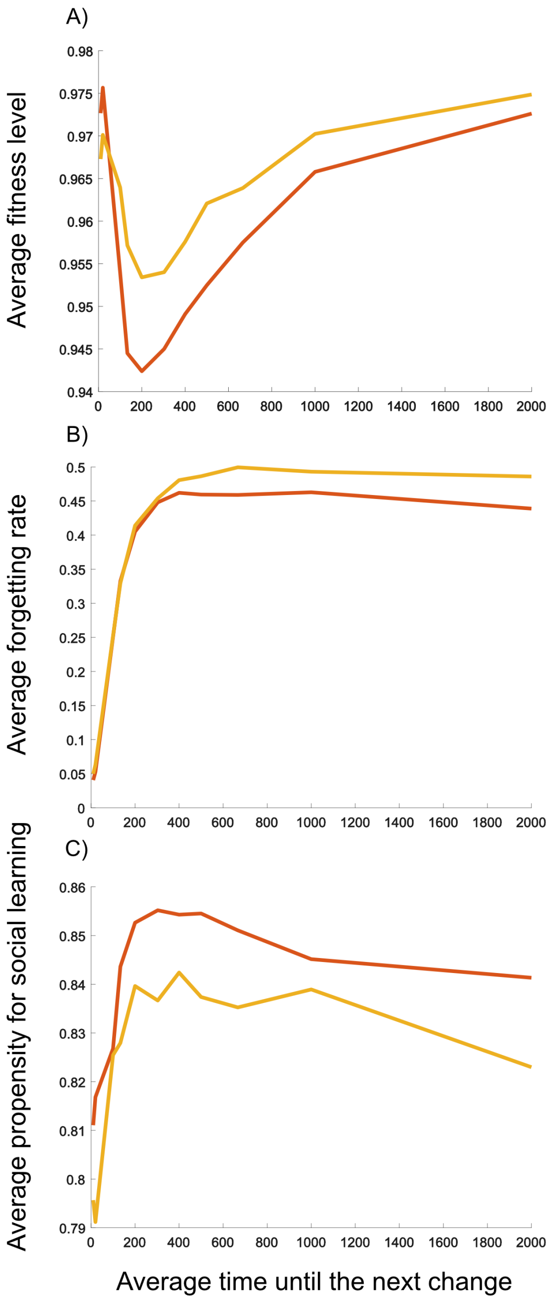

Figure I: Relationship between environmental variability and average (A) population level fitness, (B) rate of forgetting, (C) social learning propensity. Values are calculated as the average values for all individuals in the last generation (i.e. 200 time steps) of a simulation (N = 200). Population averages are further averaged over all simulations. Yellow lines correspond to simulations including vertical learning, red lines correspond to simulations without vertical learning. Results correspond to type II innovations with a softmax rule.

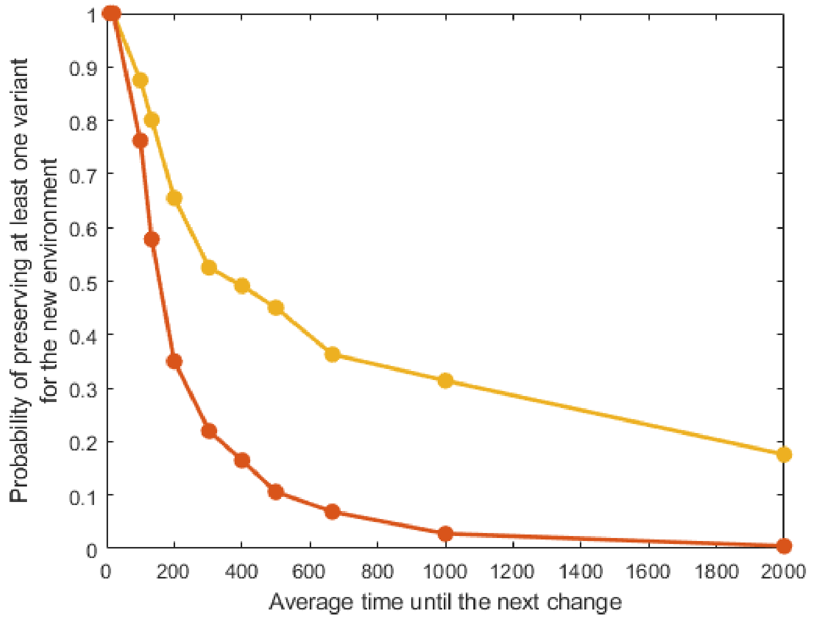

Figure J: Relationship between environmental variability and the probability of preserving at least one variant adapted to the 'alternative' environment until the next change occurs. Simulations are run until the burn-in phase and stopped once the first change occurs (before learning takes place). For each simulation it is recorded whether the population consists of at least one individual that knows about a variant adapted to the 'alternative' environment. Values are averaged over all simulations. Yellow lines correspond to simulations with vertical learning, red lines correspond to simulations without vertical learning. Results correspond to type II innovations with a softmax rule.

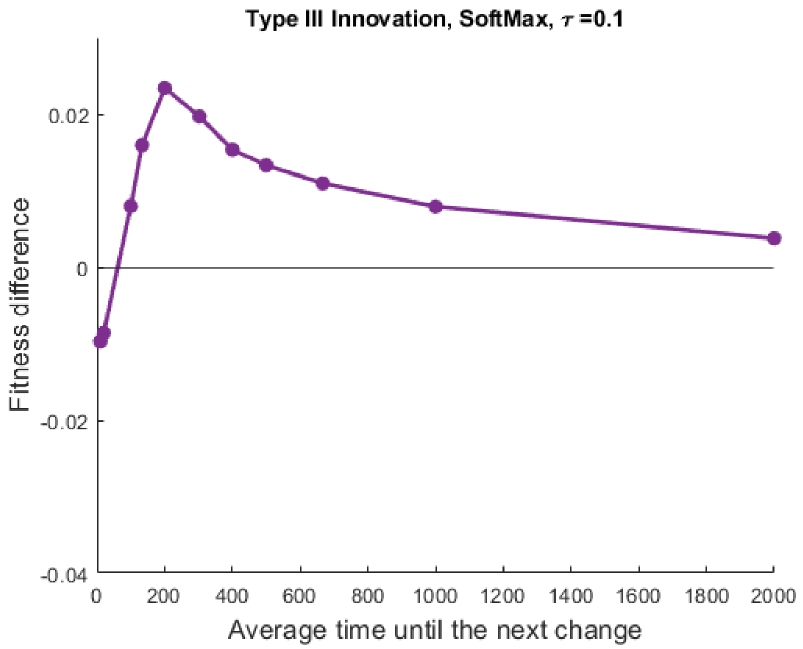

Figure K: Relationship between environmental variability and average fitness difference between populations with and without vertical learning. Values are calculated as the average fitness value received as the average expression value of variants by all individuals in the last generation (i.e. 200 time steps) of a simulation. Fitness values of populations without vertical learning are subtracted from populations exhibiting vertical learning. Population averages are further averaged over all simulations. Results correspond to type III innovations with a softmax rule.

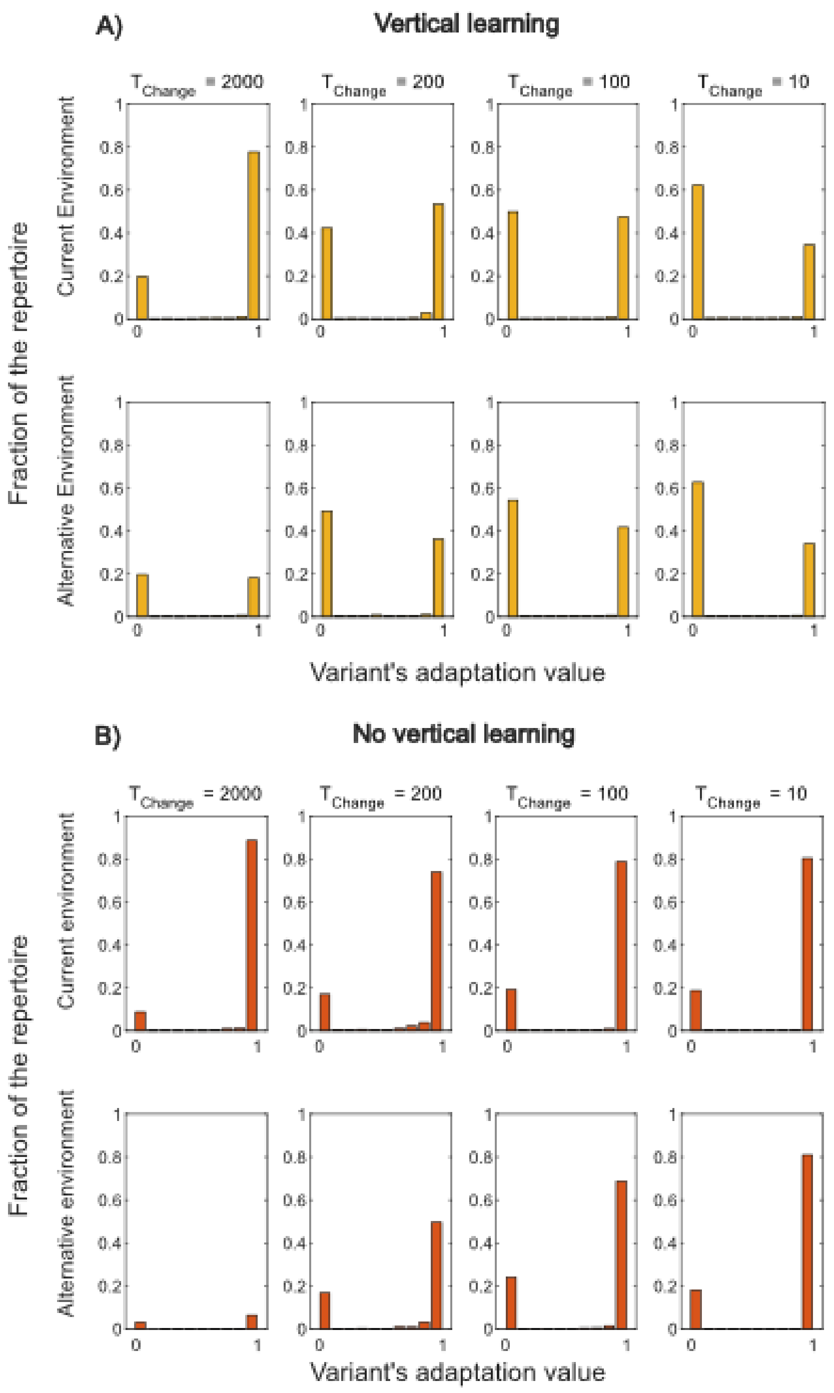

Figure L: Repertoire composition at different T_{change} values (columns) with vertical learning (A) and no vertical learning (B). Each bar shows the fraction of cultural variants that fall within a given adaptation interval, ranging from 0 (lowest adaptation value, left) to 1 (highest adaptation value, right). Fractions are calculated for single repertoires and further averaged over all simulations. The top row represents cultural variants adapted to the current environment, the bottom row represents variants adapted to the alternative environment. Results correspond to type III innovations with a softmax rule.

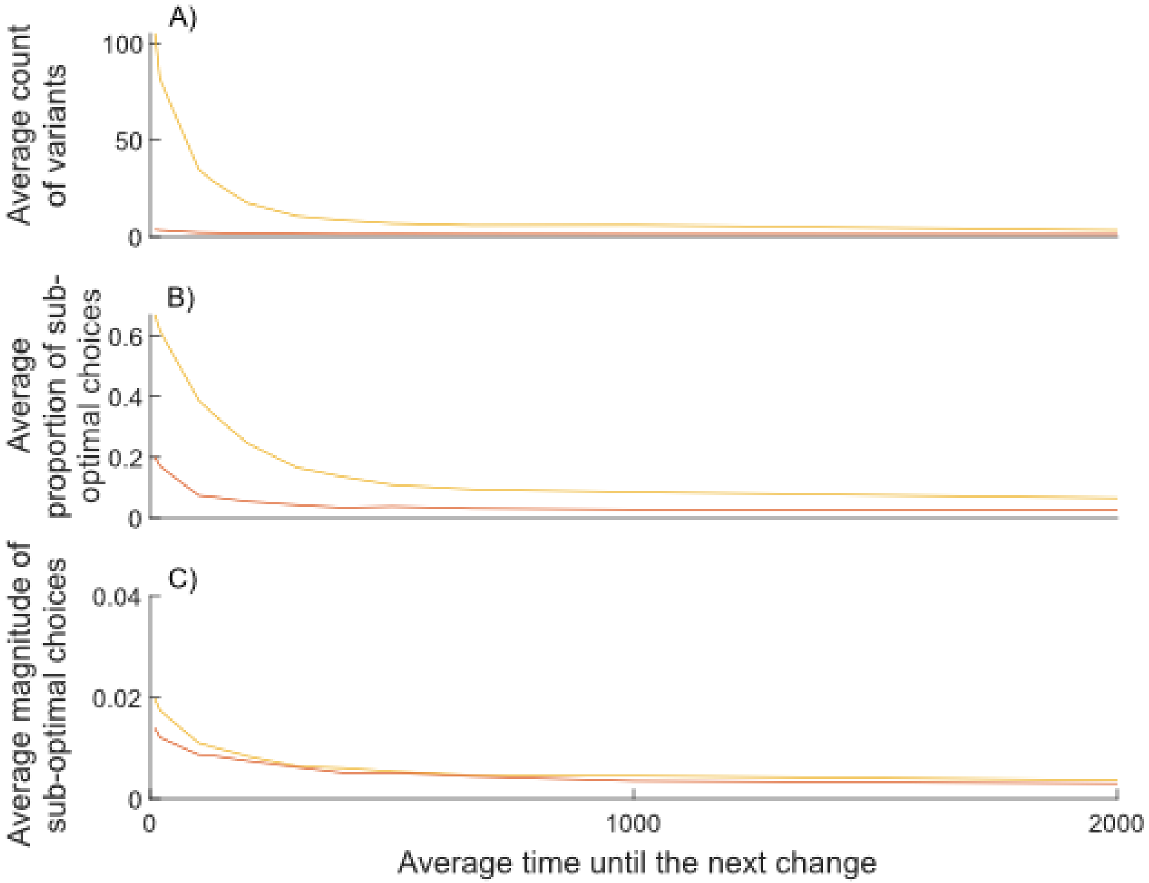

Figure M: Repertoire size and features sub-optimal variant choice at different T_{change} values (x-axis). A) Average count of variants: Values are calculated as the average variant count per individual in the last generation (i.e. 200 time steps) of a simulation. B) Average proportion of individual's sub-optimal variant: Values are calculated as the age-normalized proportion of sub-optimal variant choices of individuals living in the last generation of a simulation. C) Average magnitude of sub-optimal choice: Values are calculated as the age-normalized average distance between individual's sub-optimal variant choice and the best variant in their repertoire. Averages are calculated for individuals living in the last generation of a simulation. Population averages are further averaged over all simulations. Yellow line corresponds to the population with vertical learning, red line corresponds to the population without vertical learning. Results correspond to type III innovations with a softmax rule.

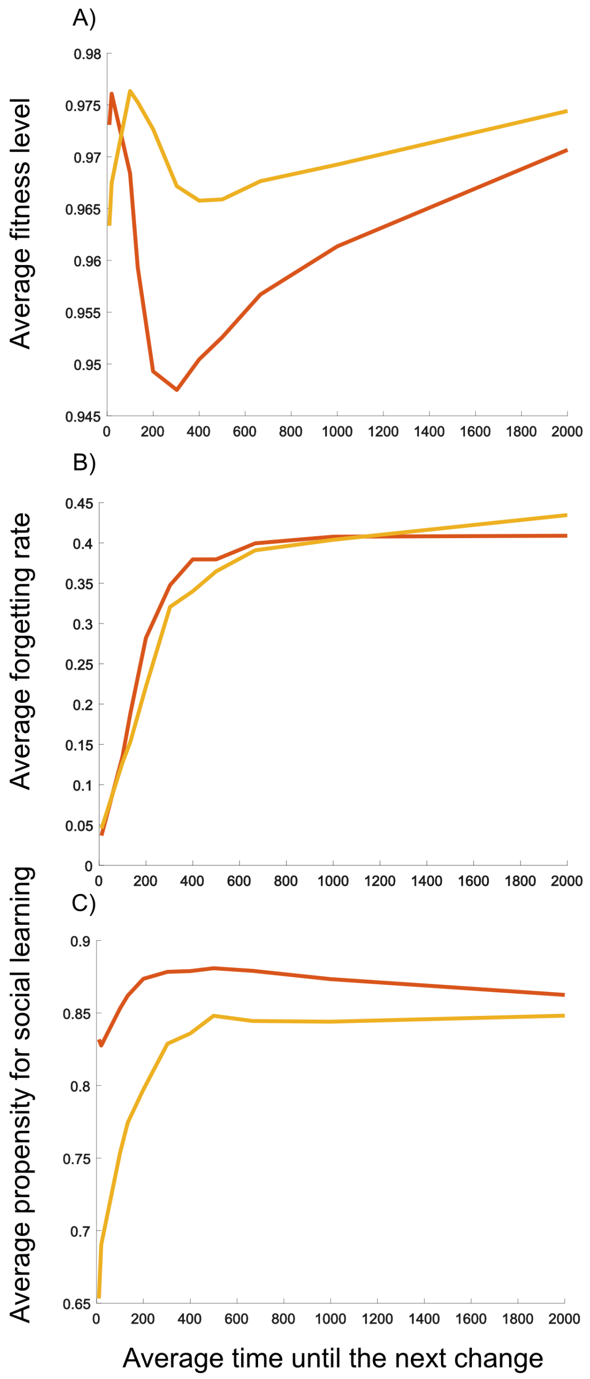

Figure N: Relationship between environmental variability and average (A) population level fitness, (B) rate of forgetting, (C) social learning propensity. Values are calculated as the average values for all individuals in the last generation (i.e. 200 time steps) of a simulation (N = 200). Population averages are further averaged over all simulations. Yellow lines correspond to simulations including vertical learning, red lines correspond to simulations without vertical learning. Results correspond to type III innovations with a softmax rule.

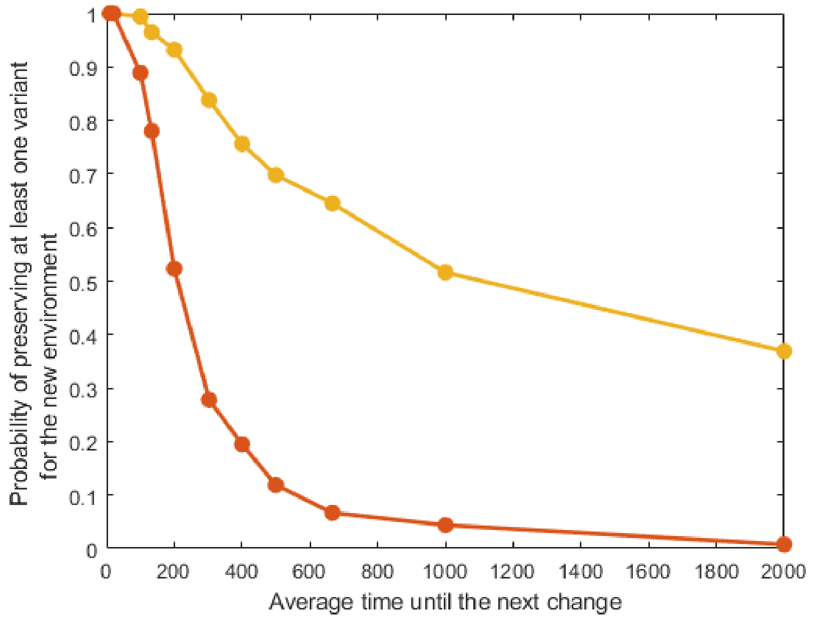

Figure O: Relationship between environmental variability and the probability of preserving at least one variant adapted to the ‘alternative’ environment until the next change occurs. Simulations are run until the burn-in phase and stopped once the first change occurs (before learning takes place). For each simulation it is recorded whether the population consists of at least one individual that knows about a variant adapted to the ‘alternative’ environment. Values are averaged over all simulations. Yellow lines correspond to simulations with vertical learning, red lines correspond to simulations without vertical learning. Results correspond to type III innovations with a softmax rule.

A

S5 Alternative innovation processes

As stated in the main text, we relax the assumption that a variant can only be adapted to one of the two considered environmental states.

In particular, we investigate the evolutionary dynamics in situations where

- the adaptation values for both environmental states are modelled as correlated, random variables, each uniformly distributed on $[0, 1]$, with correlation coefficient $\varrho = -0.9$ (see Fig. A) and
- the adaptation values are drawn uniformly from [0,1] in both states.

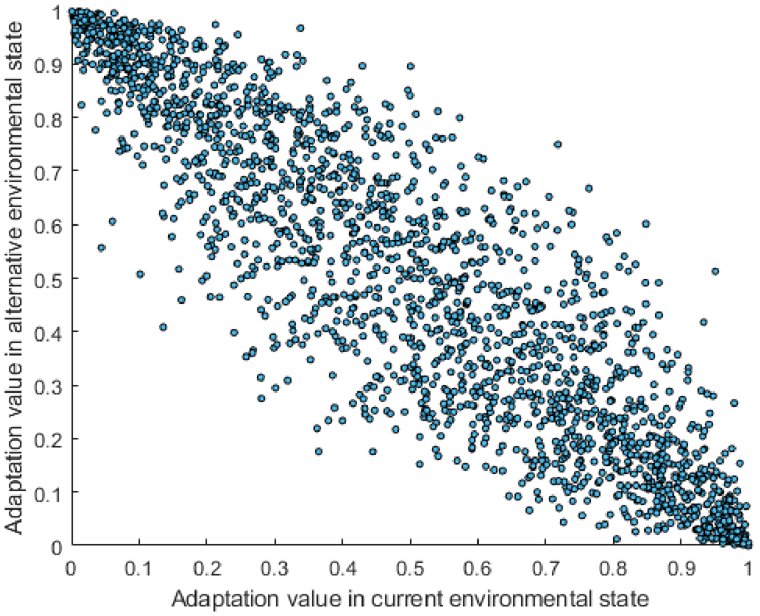

Figure A: Scatter plot of adaptation values for both environmental states when cultural variants are drawn from uniform distributions on $[0, 1]$ with correlation coefficient $\varrho = -0.9$. Each dot represents one of 2000 draws.

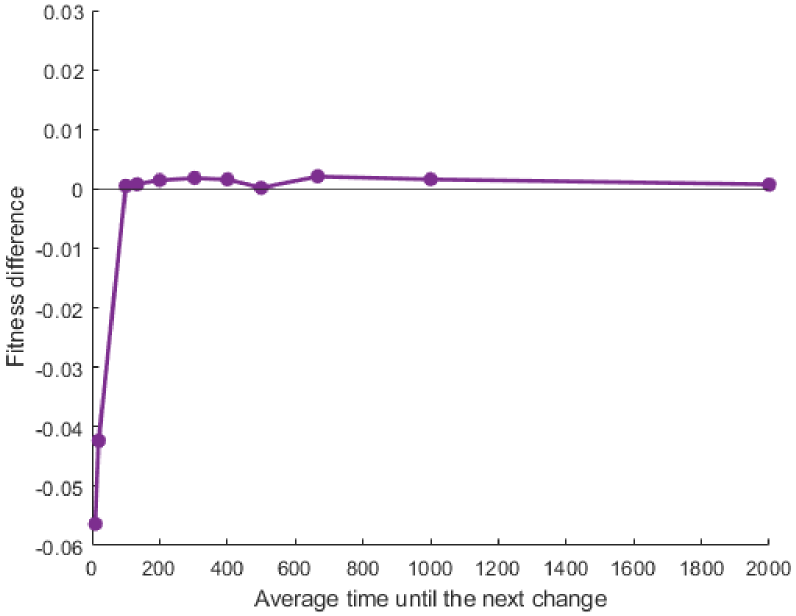

Figure B: Relationship between environmental variability and average fitness difference between populations with and without vertical learning when the adaptation values for both environmental states are modelled as correlated, random variables, each uniformly distributed on $[0, 1]$, with correlation coefficient $\varrho = -0.9$. Values are calculated as the average fitness value received as the average expression value of variants by all individuals in the last generation (i.e. 200 time steps) of a simulation. Fitness values of populations without vertical learning are subtracted from populations exhibiting vertical learning. Population averages are further averaged over all simulations.

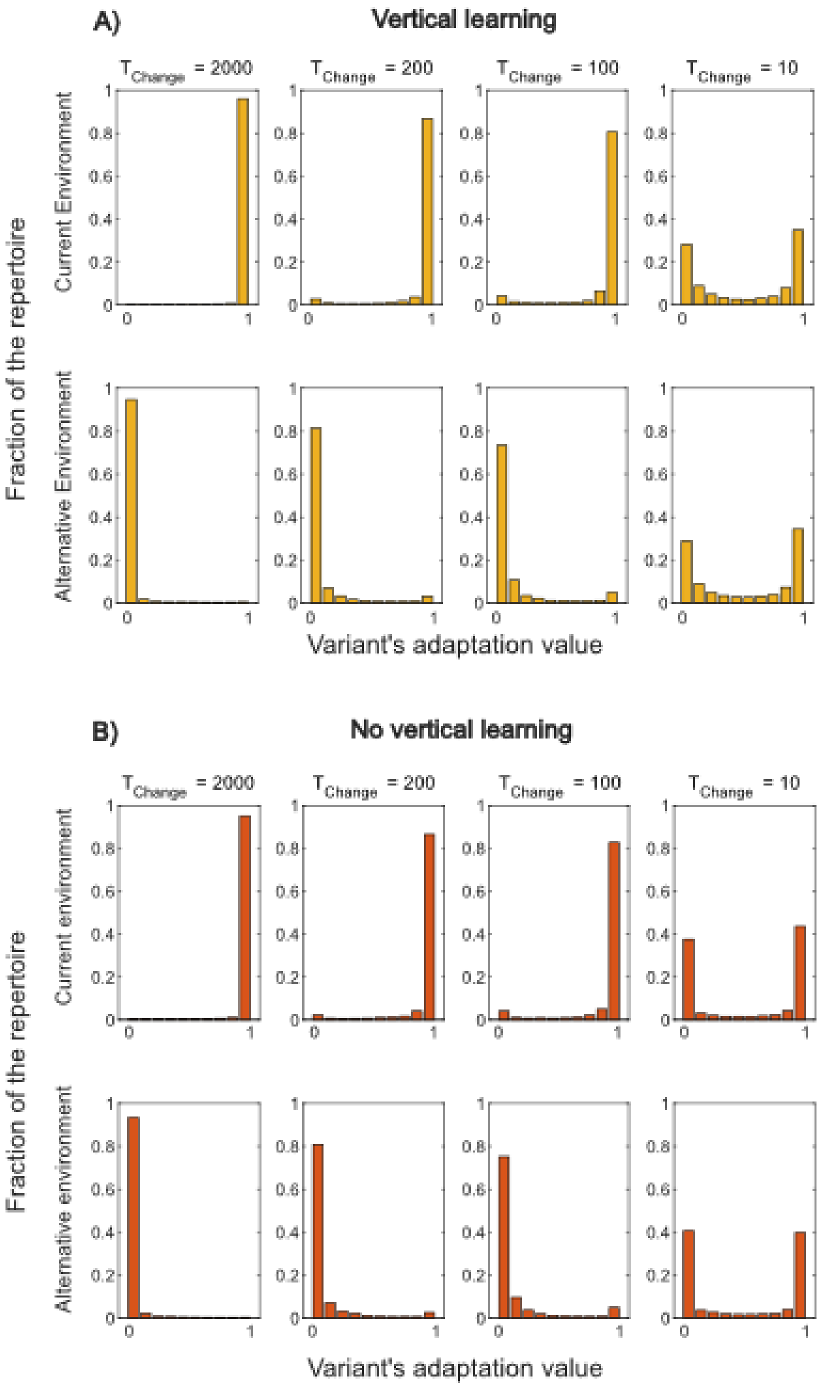

Figure C: Repertoire composition at different T_{change} values (columns) with vertical learning (A) and no vertical learning (B). Each bar shows the fraction of cultural variants that fall within a given adaptation interval, ranging from 0 (lowest adaptation value, left) to 1 (highest adaptation value, right). Fractions are calculated for single repertoires and further averaged over all simulations. The top row represents cultural variants adapted to the current environment, the bottom row represents variants adapted to the alternative environment. Results correspond to situations in which the adaptation values for both environmental states are modelled as correlated, random variables, each uniformly distributed on $[0, 1]$, with correlation coefficient $\varrho = -0.9$.

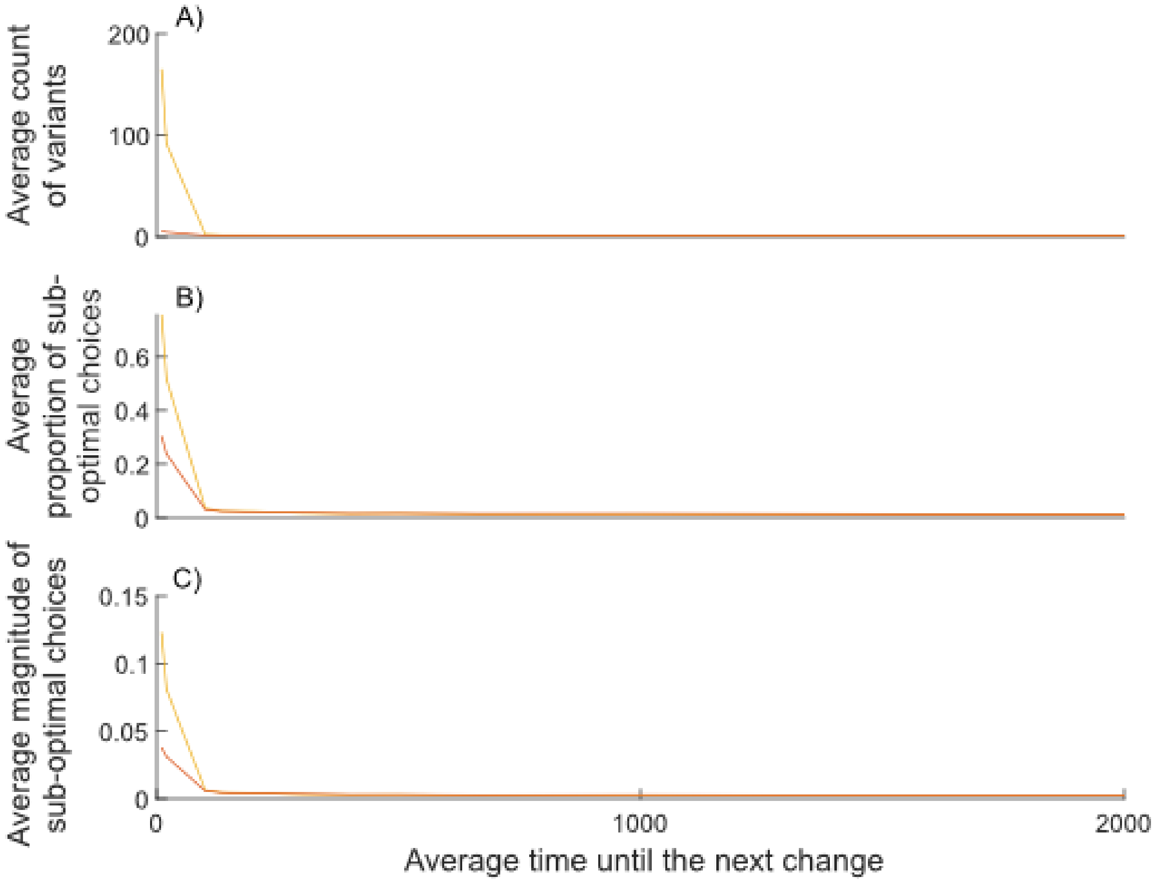

Figure D: Repertoire size and features sub-optimal variant choice at different T_{change} values (x-axis) when the adaptation values for both environmental states are modelled as correlated, random variables, each uniformly distributed on $[0, 1]$, with correlation coefficient $\varrho = -0.9$. A) Average count of variants: Values are calculated as the average variant count per individual in the last generation (i.e. 200 time steps) of a simulation. B) Average proportion of individual's sub-optimal variant: Values are calculated as the age-normalized proportion of sub-optimal variant choices of individuals living in the last generation of a simulation. C) Average magnitude of sub-optimal choice: Values are calculated as the age-normalized average distance between individual's sub-optimal variant choice and the best variant in their repertoire. Averages are calculated for individuals living in the last generation of a simulation. Population averages are further averaged over all simulations. Yellow line corresponds to the population with vertical learning, red line corresponds to the population without vertical learning.

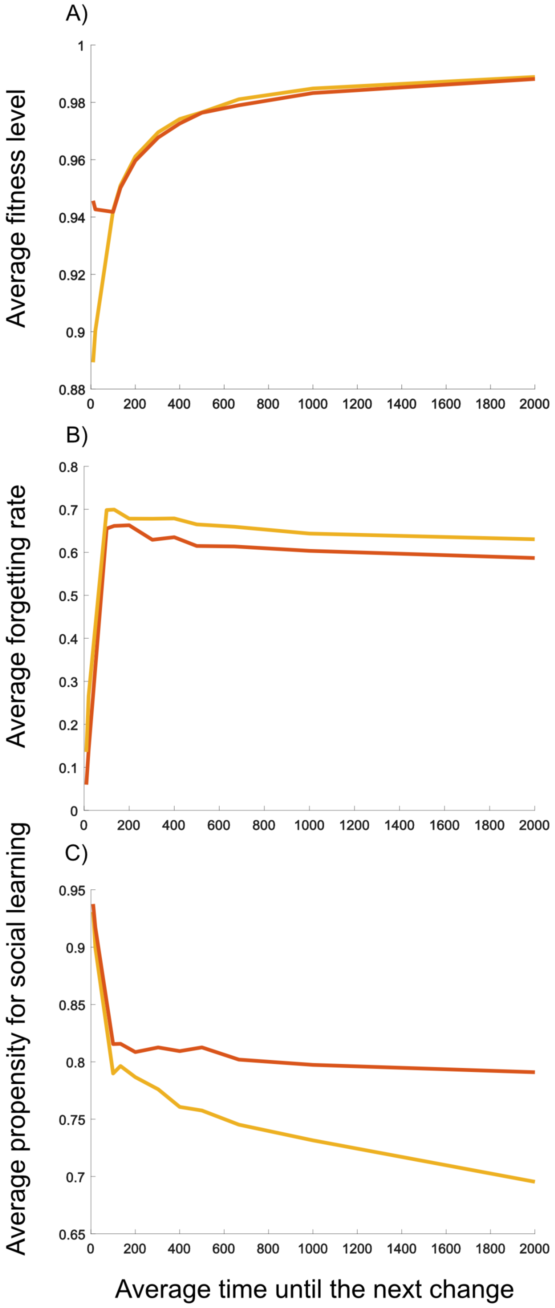

Figure E: Relationship between environmental variability and average (A) population level fitness, (B) rate of forgetting, (C) social learning propensity when the adaptation values for both environmental states are modelled as correlated, random variables, each uniformly distributed on $[0, 1]$, with correlation coefficient $\varrho = -0.9$. Values are calculated as the average values for all individuals in the last generation (i.e. 200 time steps) of a simulation (N = 200). Population averages are further averaged over all simulations. Yellow lines correspond to simulations including vertical learning, red lines correspond to simulations without vertical learning.

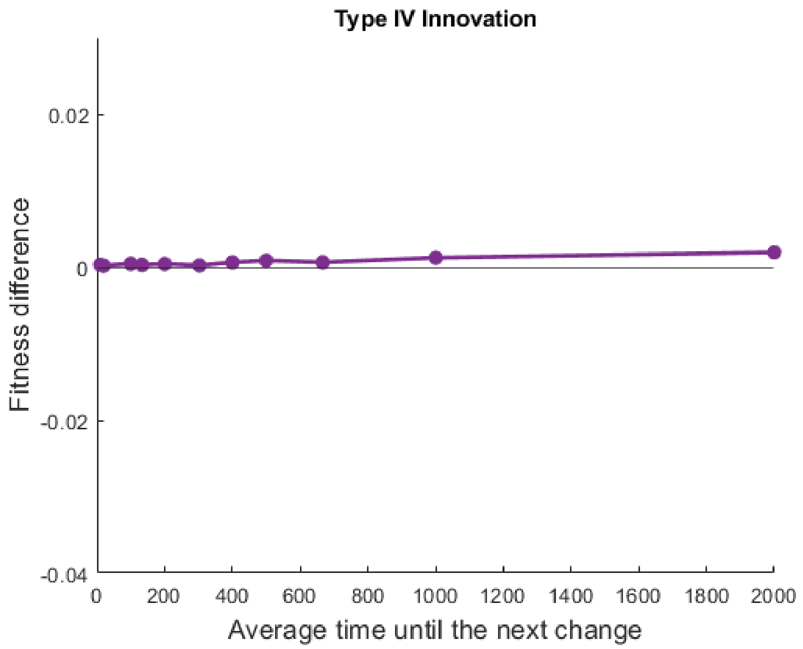

Figure F: Relationship between environmental variability and average fitness difference between populations with and without vertical learning when the adaptation values are drawn uniformly from [0,1] in both states. Values are calculated as the average fitness value received as the average expression value of variants by all individuals in the last generation (i.e. 200 time steps) of a simulation. Fitness values of populations without vertical learning are subtracted from populations exhibiting vertical learning. Population averages are further averaged over all simulations.

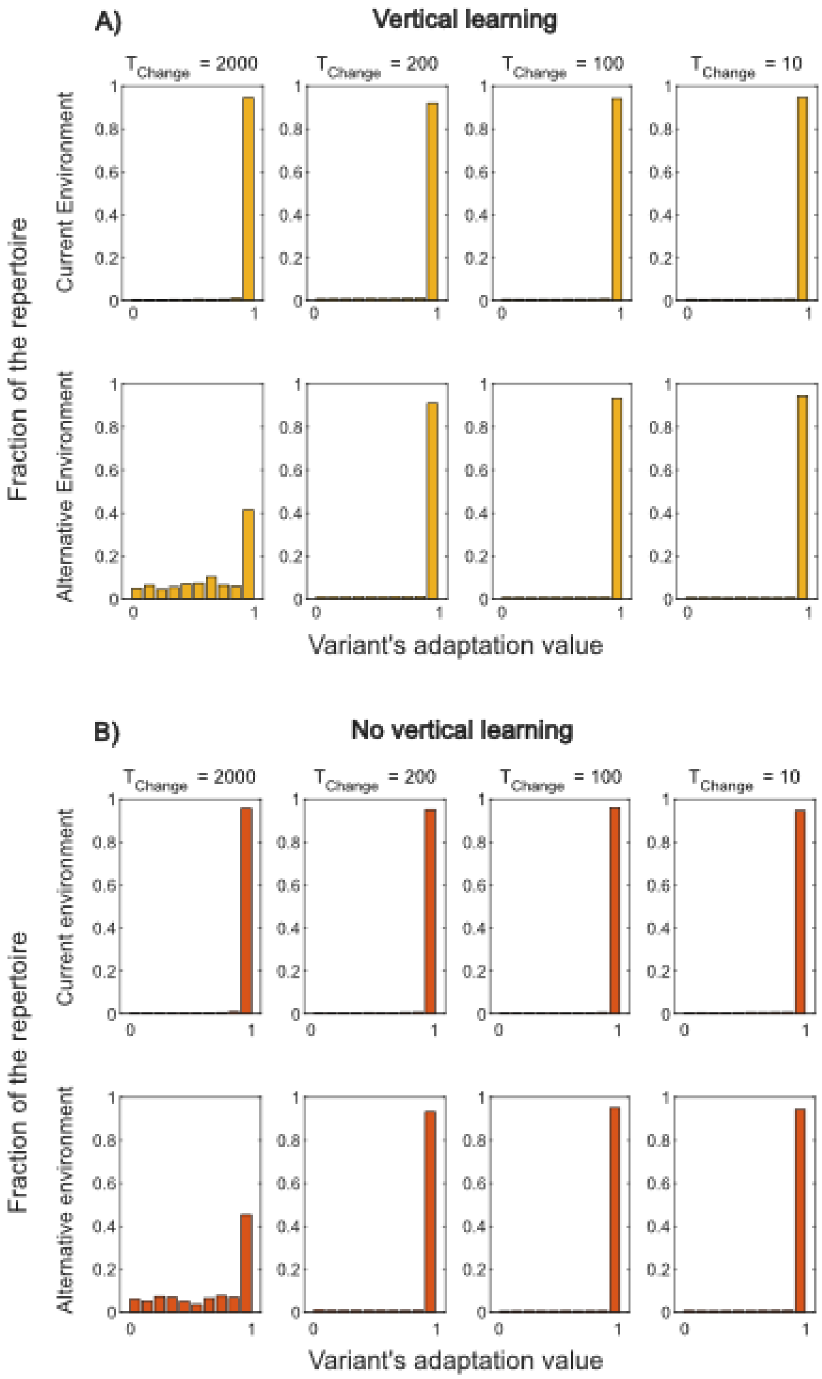

Figure G: Repertoire composition at different T_{change} values (columns) with vertical learning (A) and no vertical learning (B). Each bar shows the fraction of cultural variants that fall within a given adaptation interval, ranging from 0 (lowest adaptation value, left) to 1 (highest adaptation value, right). Fractions are calculated for single repertoires and further averaged over all simulations. The top row represents cultural variants adapted to the current environment, the bottom row represents variants adapted to the alternative environment. Results correspond to situations in which the adaptation values are drawn uniformly from [0,1] in both states.

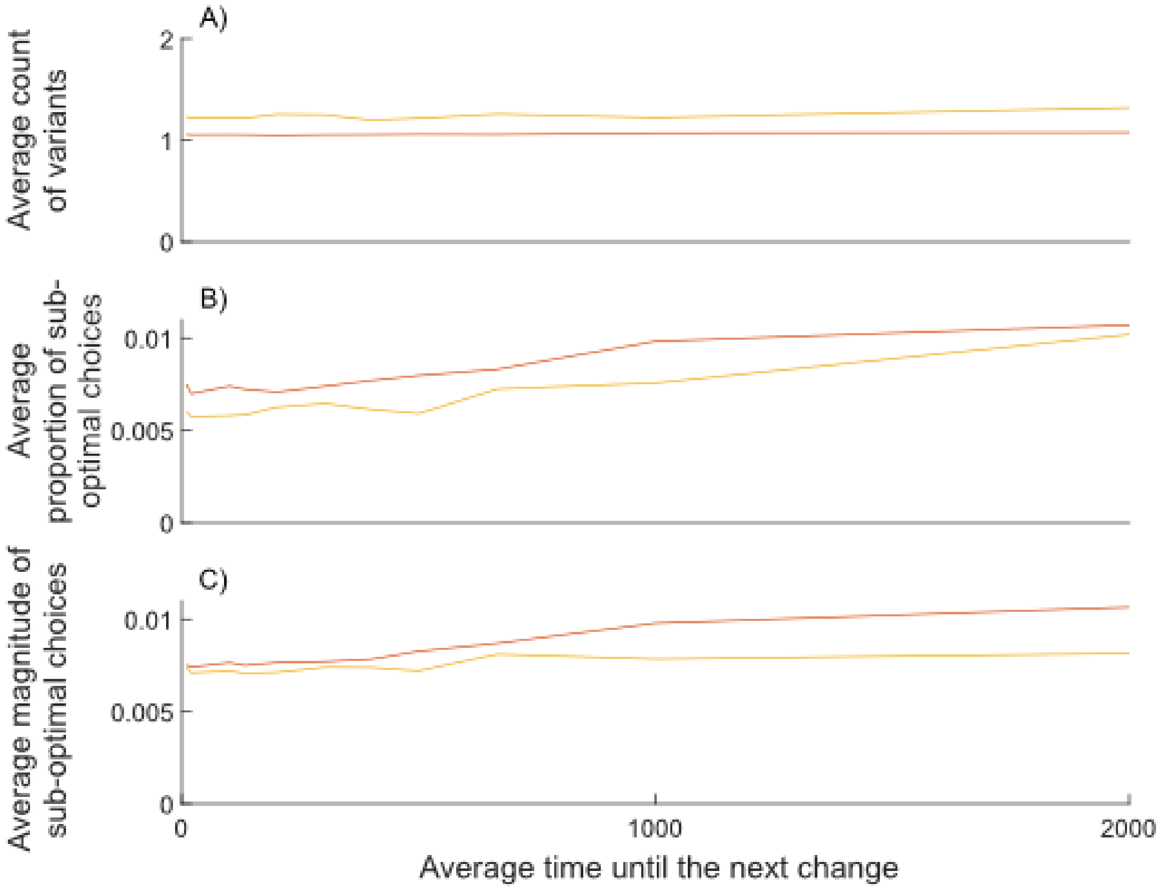

Figure H: Repertoire size and features sub-optimal variant choice at different T_{change} values (x-axis) when the adaptation values are drawn uniformly from [0,1] in both states. A) Average count of variants: Values are calculated as the average variant count per individual in the last generation (i.e. 200 time steps) of a simulation. B) Average proportion of individual's sub-optimal variant: Values are calculated as the age-normalized proportion of sub-optimal variant choices of individuals living in the last generation of a simulation. C) Average magnitude of sub-optimal choice: Values are calculated as the age-normalized average distance between individual's sub-optimal variant choice and the best variant in their repertoire. Averages are calculated for individuals living in the last generation of a simulation. Population averages are further averaged over all simulations. Yellow line corresponds to the population with vertical learning, red line corresponds to the population without vertical learning.

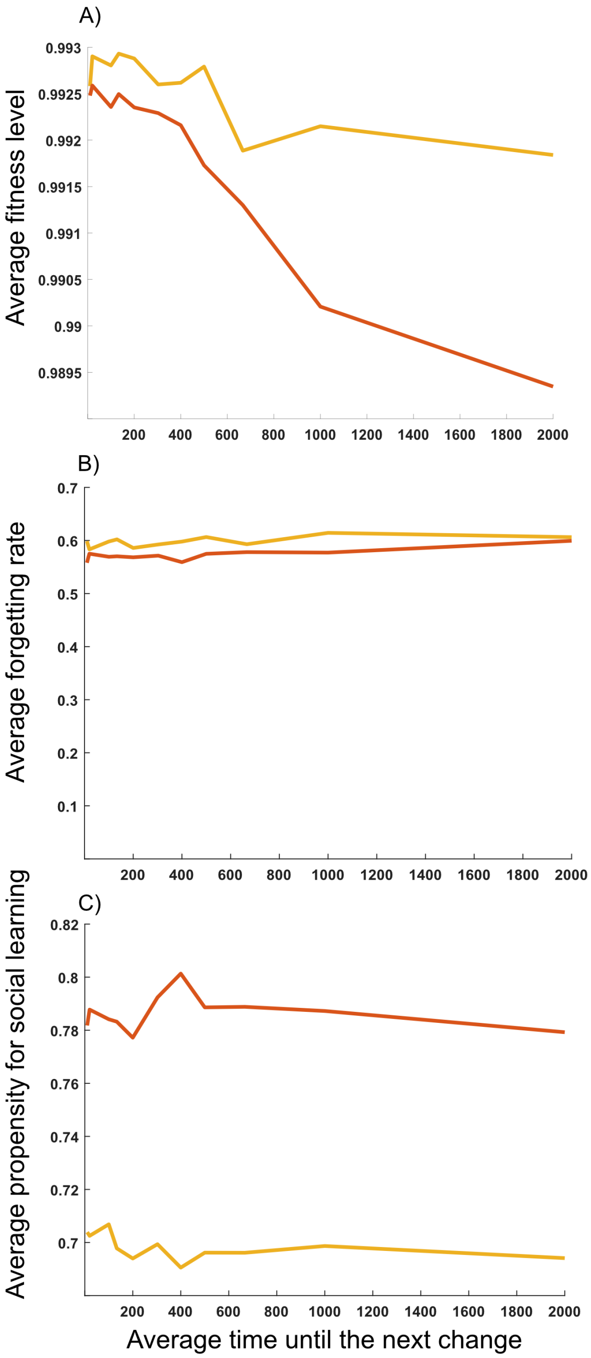

Figure I: Relationship between environmental variability and average (A) population level fitness, (B) rate of forgetting, (C) social learning propensity when the adaptation values are drawn uniformly from [0,1] in both states. Values are calculated as the average values for all individuals in the last generation (i.e. 200 time steps) of a simulation (N = 200). Population averages are further averaged over all simulations. Yellow lines correspond to simulations including vertical learning, red lines correspond to simulations without vertical learning.

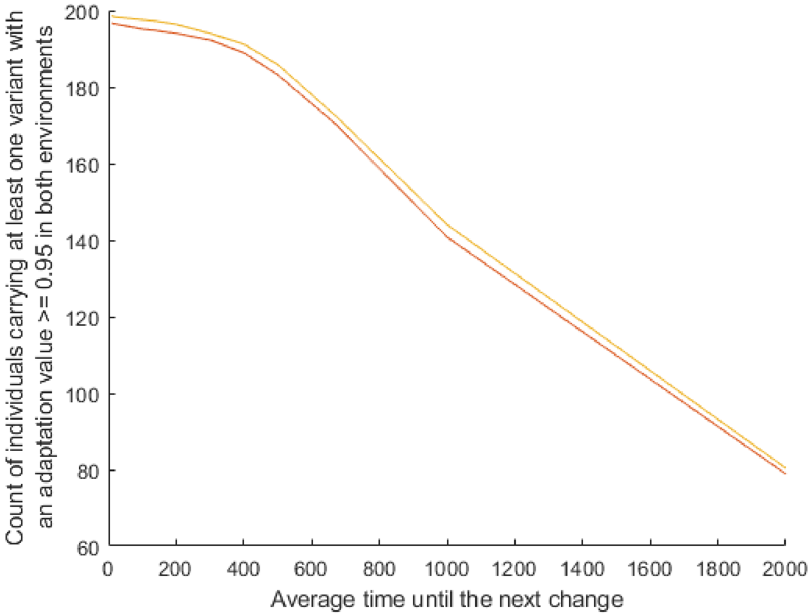

Figure J: Relationship between environmental variability and average count of individuals in population carrying a variant with an adaptation value >=0.95 in both environmental states. Results refer to situations where the adaptation values are drawn uniformly from [0,1] in both states. Values are calculated as the average values for all individuals in the last generation (i.e. 200 time steps) of a simulation (N = 200). Population averages are further averaged over all simulations. Yellow lines correspond to simulations including vertical learning, red lines correspond to simulations without vertical learning.
